# Clathrin-adaptor ratio and membrane tension regulate the flat-to-curved transition of the clathrin coat during endocytosis

**DOI:** 10.1038/s41467-018-03533-0

**Published:** 2018-03-16

**Authors:** Delia Bucher, Felix Frey, Kem A. Sochacki, Susann Kummer, Jan-Philip Bergeest, William J. Godinez, Hans-Georg Kräusslich, Karl Rohr, Justin W. Taraska, Ulrich S. Schwarz, Steeve Boulant

**Affiliations:** 1 Department of Infectious Diseases, Virology, University Hospital Heidelberg, Im Neuenheimer Feld 324, 69120 Heidelberg, Germany; 2 German Cancer Research Center (DKFZ), Im Neuenheimer Feld 581, 69120 Heidelberg, Germany; 3 BioQuant Center, Im Neuenheimer Feld 267, 69120 Heidelberg, Germany; 4 Institute for Theoretical Physics, Heidelberg University, Philosophenweg 19, 69120 Heidelberg, Germany; 5 National Heart Lung and Blood Institute, National Institutes of Health, Bethesda, MD 20892 USA; 6 Institute of Pharmacy and Molecular Biotechnology (IPMB), Department of Bioinformatics and Functional Genomics, Heidelberg University, Im Neuenheimer Feld 267, 69120 Heidelberg, Germany

## Abstract

Although essential for many cellular processes, the sequence of structural and molecular events during clathrin-mediated endocytosis remains elusive. While it was long believed that clathrin-coated pits grow with a constant curvature, it was recently suggested that clathrin first assembles to form flat structures that then bend while maintaining a constant surface area. Here, we combine correlative electron and light microscopy and mathematical growth laws to study the ultrastructural rearrangements of the clathrin coat during endocytosis in BSC-1 mammalian cells. We confirm that clathrin coats initially grow flat and demonstrate that curvature begins when around 70% of the final clathrin content is acquired. We find that this transition is marked by a change in the clathrin to clathrin-adaptor protein AP2 ratio and that membrane tension suppresses this transition. Our results support the notion that BSC-1 mammalian cells dynamically regulate the flat-to-curved transition in clathrin-mediated endocytosis by both biochemical and mechanical factors.

## Abstract (web summary)

The sequence of structural and molecular events during clathrin-mediated endocytosis is unclear. Here the authors combine correlative microscopy and simple mathematical growth laws to demonstrate that the flat patch starts to curve when around 70% of the final clathrin content is reached.

## Introduction

**Fig. 1:**
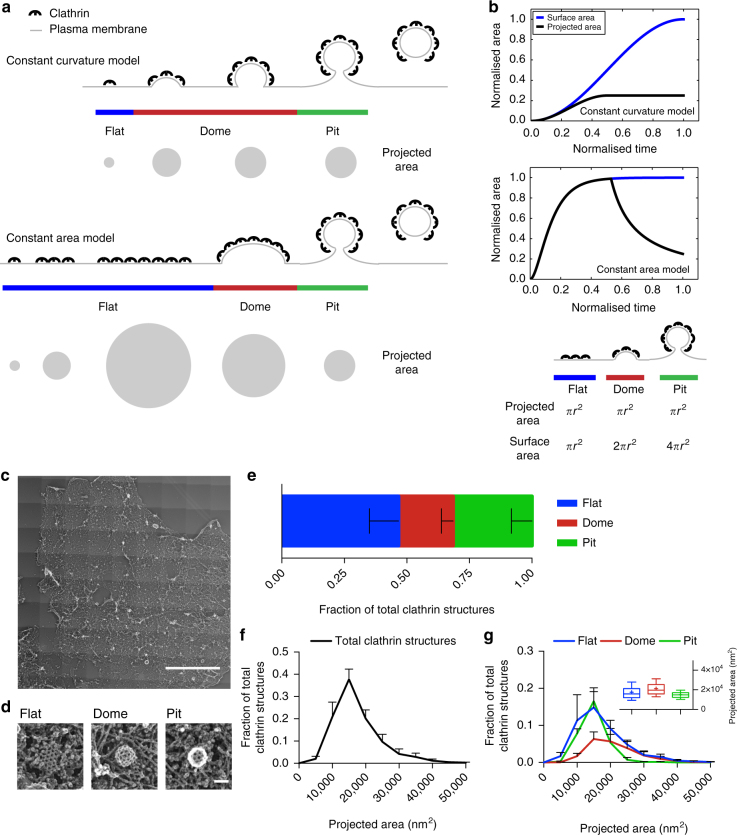
Comprehensive ultrastructural characterisation of CCS in BSC-1 cells by TEM. **a** Schematic of the constant curvature and constant area models. The stages of different curvature (flat (blue), dome (red), pit (green)) and the variation of projected area, which can be assessed during TEM imaging, is depicted for both growth models. **b** Difference between projected area (black) and surface area (blue) during the course of CCP formation according to the two models. The schematic illustrates the relationship between projected area and surface area for flat, dome (approximately a hemisphere) and pit (approximately a complete sphere) CCSs. **c** TEM of metal replica from unroofed PM, overview of whole membrane, scale bar: 10 µm. **d** Examples of flat, dome and pit structures, scale bar: 100 nm. **e** Fraction of flat (blue), dome (red) and pit (green) CCSs in whole PM of BSC-1 cell. **f** Projected area distribution of all CCSs (black) measured by TEM. **g** Projected area distribution of the different clathrin morphologies (flat, dome, pit). A box/whisker plot of the projected area is shown in the inset. Mid-line represents median, cross represents the mean and the whiskers represent the 10 and 90 percentiles. Results are calculated from three different membranes (number of CCSs per membrane: 746, 869 and 739); means with SD are shown

Clathrin-mediated endocytosis (CME) is an essential uptake pathway that relocates membrane or extracellular cargo into the cell to regulate multiple cellular functions and cell homeostasis^1^. During CME, the clathrin coat is assembled to form a clathrin-coated pit (CCP) that after dynamin-mediated scission from the plasma membrane (PM) leads to the formation of a clathrin-coated vesicle (CCV)^2^. This process is coordinated by numerous adaptor and accessory proteins^1,3^. Electron microscopy (EM) of clathrin-coated structures (CCSs) has shown the architectural complexity of the clathrin meshwork organised into hexagons and pentagons^4,5^. From this EM analysis, it was proposed that a CCV initiates as a flat clathrin lattice that is then rearranged to form a curved CCP^4,6,7^. However, for topological reasons this requires a major ultrastructural rearrangement of the clathrin lattice which appeared to be dynamically difficult and energetically costly^8–12^. For these reasons, this notion was replaced by a general belief that CCSs grow with a constant curvature (constant curvature model, Fig. 1a)^8,9,13^ and that flat CCSs are distinct from CCPs and serve different purposes^14–16^. This model was supported by the finding that purified clathrin triskelia self-assemble into curved clathrin baskets in vitro^17,18^. Recently, correlative light and electron microscopy (CLEM) analyses provided experimental evidence that CCSs first grow flat to their final size and then acquire curvature (constant area model, Fig. 1a)^19^. However, this study did not measure the dynamics of CCP formation directly, and it did not identify the cellular factors that might determine when the flat-to-curved transition occurs. Thus, a comprehensive understanding of the dynamic process of coat rearrangement, of the temporal aspects of flat-to-curved transition and of what governs this ultrastructural rearrangement during CME is still missing.

In this work, we combine simple mathematical reasoning and CLEM analysis to provide a comprehensive description of the dynamic ultrastructural rearrangement of the clathrin coat during CME. We demonstrate that CCPs indeed initially grow as flat arrays, but that their reorganisation into curved structures occurs before reaching their full clathrin content. We correlate this flat-to-curved transition with a change in the AP2/clathrin ratio and show that it is governed by biophysical properties of the PM. Our findings provide a unifying view of the dynamic process of coat rearrangement during CME and our approach constitutes a methodological framework to further study the fine-tuned spatio-temporal mechanism regulating coat assembly.

## Results

### EM analyses of CCSs do not support existing growth models

To address whether CCP formation follows the constant curvature model or the constant area model (Fig. 1a)^13^, we chose BSC-1 cells, a widely used cellular model to study CME^10,14,20^. BSC-1 cells present homogenous CME events in regard to both lifetime and intensity profiles and lack the long-lived flat clathrin-coated plaques^10,15^ (Supplementary Fig. 1 and Fig. 1c–g). Both models predict different growth profiles for the surface and projected area during CCP formation. The constant curvature model implies that the projected area will quickly be smaller than the surface area. In contrast, the constant area model implies that both projected and surface areas initially show similar growth but then the projected area should drop significantly as bending starts (Fig. 1b).

To comprehensively characterise the ultrastructural organisation of CCSs in BSC-1 cells, we performed transmission electron microscopy (TEM) of metal replicas from unroofed PMs^4,21^ (Fig. 1c). We confirmed that CCSs are not altered by the unroofing procedure using stimulated emission depletion (STED) super-resolution microscopy of intact and unroofed cells. The number and size distribution of CCSs were indeed similar between intact and unroofed cells (Supplementary Fig. 2). CCSs in TEM images of whole PM sheets were counted, categorised as flat, dome or pit structures (Fig. 1d, e) and their size was measured as projected area (Fig. 1a, f, g). For the constant curvature model, we would expect no flat structures at all and no dome structures that exceed the projected area of pits (Fig. 1a, b). In contrast, our EM data reveal that around 50% of the CCSs in BSC-1 correspond to flat CCSs (Fig. 1e) and that a large fraction of the flat and dome structures has a projected area larger than the projected area of the pits (Fig. 1g). Since BSC-1 cells do not have clathrin-coated plaques^10,15^, these results demonstrate that the constant curvature model cannot explain the CCS size distribution, in agreement with the recent results by Avinoam et al^19^. Since the existence of flat CCSs seems to argue in favour of the constant area model, we would expect that some flat structures have the same projected area as the surface area of fully formed pits (Fig. 1a, b). Since the surface area of a spherical pit (4*πr*^2^, Fig. 1b) is four times larger than its projected area (*πr*^2^, Fig. 1b), we would expect the mature flat structures to have around four times the projected area of pits (Fig. 1b). Instead, we found no flat structures at all with a projected area four times larger than the mean projected area of CCP (Fig. 1g). Additionally, the constant area model would imply that the projected area of a dome structure (which resembles a hemisphere) is reduced by a factor of two when converting to a CCP. Instead, we found only a slight increase of the mean projected area of domes compared to pits (Fig. 1g, distribution and inset box/whiskers). Together, these observations argue against the constant area model.

**Fig. 2:**
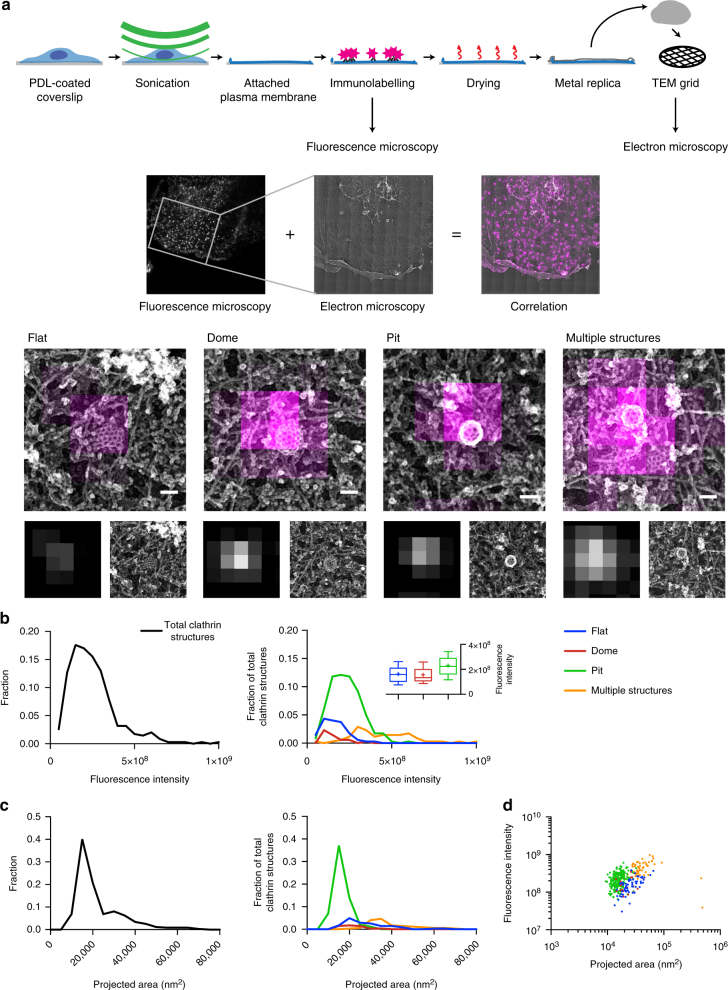
CLEM of CCSs. **a** Schematic representation of the CLEM approach (upper part). Cells growing on poly-d-lysine (PDL)-coated coverslips were unroofed by sonication. Attached PMs were immunostained and imaged using FM. Samples were then critical-point dried and a metal replica was created and lifted from the sample onto a TEM grid for imaging in TEM. (Middle part) The FM and EM pictures were then correlated to combine their information (see Methods). Unroofed PMs were immunostained using an anti-clathrin heavy chains antibody. The white inset box represents the area observed by TEM. (Lower part) Examples of flat, dome, pit and multiple structures; top panel: CLEM, lower left: FM lower right: TEM; scale bar: 100 nm. **b** Fluorescence intensity distribution (clathrin heavy chain antibody, X22) of all CCSs (black line, left panel) and of flat (blue), dome (red), pit (green) CCSs and multiple structures which cannot be distinguished by fluorescence microscopy (orange) (right panel). A box/whisker plot of the fluorescence intensity is shown in the inset. Mid-line represents median, cross represents the mean and the whiskers represent the 10 and 90 percentiles. **c** Projected area distribution of all CCSs (black line, left panel) and of the different clathrin morphologies (right panel). **d** Correlation of size and fluorescence intensity of all CCSs sorted by their different morphologies. Graphs show one representative CLEM result with a total of 347 CCSs from one PM

To further challenge the two growth models, we used a CLEM approach^21,22^ (Fig. 2a). BSC-1 cells were immunostained with a clathrin heavy chain antibody and the fluorescence intensity of CCSs (Fig. 2b) was correlated to their size and ultrastructural organisation measured using TEM of metal replicas (Fig. 2c, d). CCSs were classified according to their ultrastructural organisation observed by TEM as flat, dome and pit structures. Some of the CCSs could be separated and identified by TEM but not by fluorescence microscopy (FM) due to its lower resolution (i.e., multiple CCSs appearing as one fluorescent object). We classified them as “multiple structures” (Fig. 2a, lower panel) and excluded them from further analysis. For the constant curvature model, we would expect the intensity to increase with increasing contact angle (Fig. 1a, b). In case of the constant area model, we would expect equal intensity for the largest flat, dome as well as all pit structures (Fig. 1a, b). However, our CLEM analysis clearly revealed that flat and dome structures have similar fluorescence intensities while pits tend to display higher fluorescence intensities (Fig. 2b, d). These flat CCSs had a mean fluorescence intensity of around 70% of the pits (Fig. 2b box/whiskers; mean fluorescence intensity for flat CCSs: 1.6 × 10^8^; pits: 2.3 × 10^8^). In conclusion, our TEM and CLEM analyses argue that neither of the proposed growth models fully explain the observed ultrastructural distribution and corresponding fluorescence intensities of CCSs in BSC-1 cells.

### Clathrin lattice bends before reaching full clathrin content

**Fig. 3:**
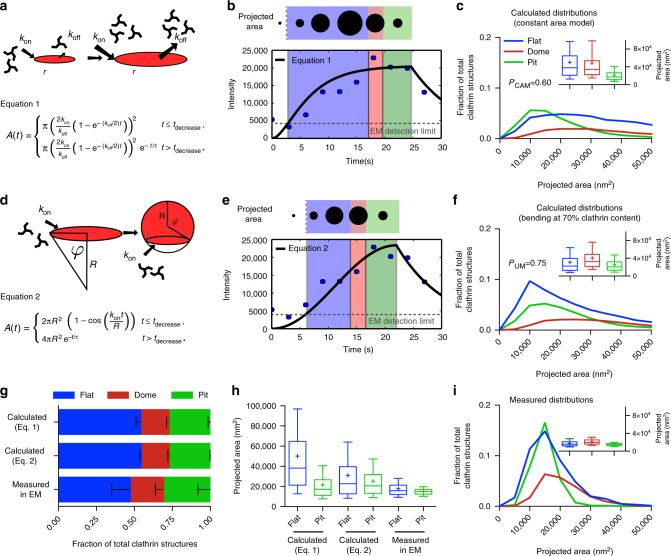
Mathematical modelling of CCS growth from intensity profiles of individual CME events. **a** Mathematical representation of the constant area model, flat-to-curved transition happens at the time when clathrin reaches its final content. **b** Example of a clathrin intensity track fitted by the constant area model. Blue dots represent measured intensity of a single CME event; black line represents the fit with Eq. 1, dashed grey line marks the EM detection limit. The schematic on the top illustrates the calculated projected area and assigned curvature, flat (blue), dome (red) and pit (green). **c** Calculated projected area and curvature distributions of the CCSs according to the constant area model for 4927 FM tracks of 4 different cells. *P*-value of Welch’s *t*-test to compare the predicted to the measured distribution in **i**. A box/whisker plot of the projected area is shown in the inset. Mid-line represents median, cross represents the mean and the whiskers represent the 10 and 90 percentiles. **d** Mathematical representation of the updated growth model where a flat clathrin patch grows and the flat-to-curved transition happens before reaching the final clathrin content. ** e**, **f** Same as **b**, **c** but using Eq. 2. **g** Comparison of the predicted ratio of flat, dome and pit structures from both growth model (Eq. 1 (**a**) and Eq. 2 (**d**)) and the distribution obtained from TEM imaging. Results are calculated for 4927 FM tracks of 4 different cells; means with SD are shown. **h** Direct comparison of the projected area distribution of flat and pit structures calculated by Eqs. 1 and 2 as well as measured in EM, box/whisker plot. **i** Measured projected area and curvature distributions of the CCSs from TEM data as shown in Fig. 1

Although EM of metal replicas is a high-resolution microscopy technique revealing detailed information about the size and ultrastructure of CCSs, it only provides snapshots of the dynamic process of CCP assembly^23^. In contrast, live FM of CME mostly allows the characterisation of the dynamics of different proteins during the formation of CCSs but does not provide ultrastructural information^9^. To obtain a more comprehensive dynamical picture, we used mathematical modelling of clathrin growth behaviour to combine the ultrastructural information from EM with the dynamic information obtained from total internal reflection fluorescence (TIRF) microscopy of fluorescently tagged clathrin light chain (CLC). Our approach is to estimate the morphology distribution of CCSs by fitting growth curves to individual CME fluorescence profiles. As the formation of CCS is a complex process with numerous yet unknown variables, here the modelling approach is used only to combine and compare the different data sets (FM, TEM and CLEM), with minimal assumptions on the underlying mechanisms. To correlate the temporal information of CCS dynamics obtained by live FM to the size information of these CCSs obtained by TEM, we used CLEM to convert the fluorescence signal into an estimate for the surface area (see Supplementary Information). Since we found a substantial number (around 50%) of flat CCSs with projected areas similar to pits (Fig. 1e, g), we rule out the possibility that the constant curvature model might be the dominant growth behaviour in BSC-1 cells. We therefore start by describing the growth of CCSs according to the constant area model. For this, CCSs first initiate as flat circular planar discs that grow to a finite size before bending (Fig. 3a). We assume growth of a CCS to be possible only at its edge, because incorporation of new triskelia in the area is expected to be energetically and topologically unfavourable. To avoid explosive growth, however, this process has to be balanced by another process. Geometrically the simplest possible model is that growth is limited by a process coupled to the area of the planar disc. Dissociation over the area would be such a process, but alternatively one could think of negative biochemical feedback increasing in proportion to the area (Fig. 3a and Supplementary Information). By fitting the resulting growth equation and assigning the three different morphologies to the individual intensity profiles obtained by TIRF microscopy (Fig. 3b and Supplementary Fig. 1g–i), we could calculate the size and morphology distribution of CCSs as predicted by the constant area model (Fig. 3c). As we are following the constant area model, CCSs grow flat to their final size which is represented by the plateau phase of the growth curves (blue area in Fig. 3b). Within the constant area model, the ratio of domes and pits is directly proportional to their transformation dynamics. We exploited the distribution of domes and pits observed in EM (40:60%, respectively) (Fig. 1e and in Avinoam et al.^19^), to define the time necessary to ultrastructurally rearrange a flat CCS to dome (red area) and a dome to CCP (green area), 40% and 60%, respectively (Fig. 3b and see Supplementary information). To calculate the size and morphology distribution of CCSs, we have to neglect the predicted objects for which sizes fall under the detection limit of TEM which we have determined as being the smallest object that can be confidently identified as clathrin coat by TEM (Fig. 3b, dashed line). This ensures that both the calculated and measured data sets are similarly restricted. Comparison of the calculated distribution to the acquired EM data reveals that the ratio between the flat, dome and pit structures is biased toward flat structures compared to the EM data (Fig. 3g). Additionally, the means of the predicted size distributions of flat and pit structures are clearly separated with a shift of the flat projected area towards bigger sizes (Fig. 3c, i, box/whiskers and Fig. 3h). In agreement with our TEM and CLEM results, our mathematical modelling approach thus demonstrates that the constant area model does not correctly describe the assembly process of CCPs in BSC-1 cells.

Given the high proportion of flat structures (Fig. 1e), we reasoned that CCSs start as flat structures and then acquire curvature before reaching the full clathrin content (Fig. 3d and Supplementary Information). In contrast to the constant area model (Fig. 3a), now the system has an intrinsic mechanism to stop growth, namely formation of a sphere, and therefore needs no balancing mechanism (no regulation by the area) besides the regulation by curvature in a minimal model. As we previously observed that the clathrin content of flat structures represents 70% of the clathrin content of CCPs (Fig. 2b, box/whiskers), we reasoned that transition from flat to dome occurs at 70% of their final clathrin content (Fig. 3e, blue area). Similar to the constant area model we defined conversion to domes (red area) and pits (green area) according to their relative ratio observed in TEM. The resulting growth equation was again fitted to the individual intensity profiles of CME events (Fig. 3e) (Supplementary Information). To test the different models we compare them on the level of size and morphology distributions (instead of single trajectories). Our approach therefore represents the typical statistics of the system and is not meant to describe single trajectories that are prone to depend strongly on molecular details. As a matter of fact, the calculated ratio between the flat, dome and pit structures is similar as before and biased towards flat structures (Fig. 3g), but in contrast now the calculated size and morphology distribution fit the EM data better than the distribution according to the constant area model. The means of the predicted projected area of both the flat and pit structures have similar sizes (Fig. 3f, h, box/whiskers). These findings strongly support a model where assembly of a CCP initiates flat and then acquires curvature at around 70% of its final clathrin content.

### The AP2/clathrin ratio governs the onset of coat curvature

**Fig. 4:**
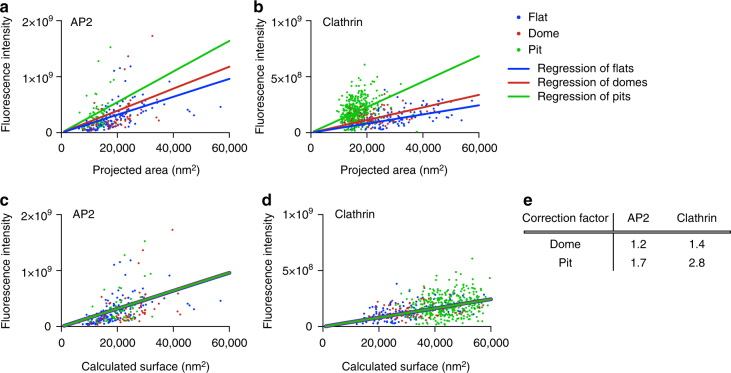
The relative amount of AP2 and clathrin molecules per surface unit of a CCS is curvature dependent. CLEM analysis of CCSs labelled with AP2-eGFP (**a**) or clathrin heavy chain antibody (**b**). Flat (blue), dome (red) and pit (green). Lines in the corresponding colour show linear regression of the projected area and of the fluorescence intensity. CLEM analysis of CCS corrected according to the regression of flat structures labelled with AP2-eGFP (**c**) or clathrin heavy chain antibody (**d**). Projected areas of dome and pit structures of the CLEM analysis were multiplied by a correction factor to fit the linear regression of flat CCS. Lines in the corresponding colour show linear regression of the calculated surface and the fluorescence intensity. **e** Table shows correction factors for dome and pit structures for AP2-eGFP or clathrin heavy chain labelling used in (**c**) and (**d**)

The flat-to-curved transition of a CCS requires major ultrastructural reorganisation of the coat^11^. To acquire curvature, according to Euler’s theorem the hexagonal organisation of the clathrin triskelia needs to reorganise into a polyhedral assembly including 12 pentagons^11^. The clathrin lattice in flat structures is mostly composed of hexagons^4,5^. Although it has been shown using fluorescence recovery after photobleaching that the clathrin coat is highly dynamic, which is a prerequisite for such rearrangement^19,24,25^, it is still puzzling what regulates the organisation of triskelia in the coat and what coordinates the flat-to-curved transition. It was proposed before that the ratio of the adaptor AP2 to clathrin changes within the growth of CCPs^22,26,27^. Therefore, we correlated the relative amount of AP2 and the ultrastructural organisation of CCSs. We performed CLEM analysis using BSC-1 cells expressing AP2 fused to green fluorescent protein (GFP; Fig. 4a) and correlated these results to clathrin immunostaining CLEM (Fig. 4b). To find the relationship between fluorescence intensity and the surface of CCSs, the measured projected area needs to be corrected for the curvature to obtain the surface area of the CCS (Fig. 1b). For flat structures, projected area and surface area are identical, and thus we used the linear regression of flat coats as a reference to correct the projected area of both domes and pits. Assuming the geometry of a hemisphere for domes and an almost complete sphere for pits, we expect a correction factor of ≤2 for domes and 2 < × ≤ 4 for pit structures if the relationship between fluorescence intensity and surface area is independent of curvature (Fig. 1b). The correction factors inferred from clathrin CLEM for domes and pits were 1.4 and 2.8, respectively, which are within the expected ranges (Figs. 4d, e and 1b). Strikingly, the correction factors for AP2 CLEM were smaller than expected, especially for the pit structures (domes: 1.2; pits: 1.7) (Fig. 4c, e). This reveals that the AP2/clathrin ratio in a CCS differs depending on its curvature and that this ratio is reduced within the coat as curvature increases.

**Fig. 5:**
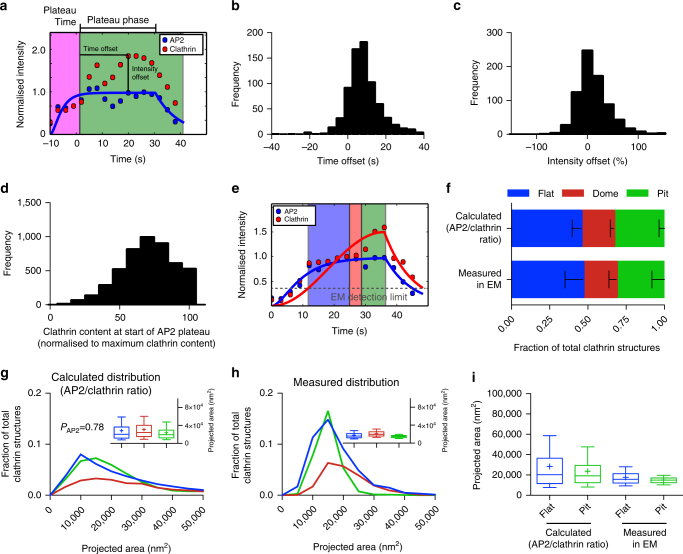
Change in the AP2/clathrin ratio is associated with flat-to-curved transition. **a** Example of an AP2 (blue) and clathrin (red) intensity profile from an individual CME event. The AP2 profile was fitted to Eq. 1 to find the time when AP2 signal plateaus. For more information see Supplementary Information. The fluorescence intensity of AP2 and clathrin was normalised to the fluorescence intensity of the time when the fitted AP2 profile reaches its plateau. Time offset (difference between the time AP2 plateaus and clathrin reaches its maximum intensity) and intensity offset (excess of maximal clathrin signal over AP2 maximum intensity) are indicated in the profiles. Quantification of the time offset (**b**) and the intensity offset (**c**) for 754 tracks of one single cell. **d** Quantification of the clathrin content at the time when AP2 reaches its plateau from 4927 FM tracks. The clathrin signal was normalised to the maximal clathrin signal in each track. **e** Example of an AP2 (blue) and clathrin (red) profile fitted to Eqs. 1 and 2, respectively. These fits were used to calculate the size and curvature distributions of the CCS in (**f**, **g**). For more information see Supplementary Information. **f** Comparison of the calculated ratio of flat, dome and pit structures to the measured ratio in TEM. Results calculated from 4927 FM tracks from 4 different cells; means with SD are shown. **g** Calculated projected area of the CCSs using a growth model where the flat-to-curved transition corroborates with the change of clathrin/AP2 ratio (when AP2 signal reaches its plateau phase) for 4927 FM tracks of 4 different cells. *P*-value of Welch’s *t*-test to compare the predicted to the measured distribution in (**h**). A box/whisker plot of the projected area is shown in the inset. **h** Measured projected area and curvature distributions of the CCSs from TEM data as shown in Fig. 1. A box/whisker plot of the projected area is shown in the inset. **i** Direct comparison of the projected area distribution of flat and pit structures calculated according to the AP2/clathrin ratio as well as measured in EM, box/whisker plot

As the AP2/clathrin ratio depends on the curvature of CCS (Fig. 4) and as AP2 partitions at different nanoscale zones in relation to the edge of the clathrin lattice during the different stages of CCS formation^22^, we hypothesised that the change in the AP2/clathrin ratio correlates with the stage at which a flat CCS bends to form a CCP. Using cells expressing both AP2 fused to GFP and CLC fused to the fluorescent protein tdtomato, we analysed the intensity profiles of AP2 and CLC during CME. While AP2 profiles often show a distinct plateau phase, the intensity of CLC continues to increase until the end of an endocytic event (Fig. 5a and Supplementary Fig. 1). By normalising the fluorescence intensities of AP2 as well as CLC to the time point when the AP2 signal plateaus, we can calculate the time offset between the time AP2 signal reaches its plateau and the time point CLC reaches its maximal intensity (Fig. 5a, b). Similarly, we defined the intensity offset of clathrin over AP2 (Fig. 5a, c). We found that the time offset was around 10 s (Fig. 5b) and that the intensity offset of clathrin over AP2 was around 15% (Fig. 5c). We hypothesised that the time point when AP2 reaches its plateau and therefore the AP2/clathrin ratio changes marks the starting point of bending. We performed another round of CCP assembly modelling, this time using both AP2 and CLC intensity profiles and defining the time point of flat-to-curved transition when AP2 reaches its plateau phase (Fig. 5e and Supplementary Information). At this precise time, the mean clathrin content reached around 70% of its maximal value (Fig. 5d). Using these new parameters, the predicted ratio of flat, dome and pit structures perfectly matched the EM data (Fig. 5f) and the means of the predicted projected area of both the flat and pit CCSs have similar sizes (Fig. 5g–h, box/whiskers and 5i). This AP2/clathrin ratio model better resembles the parameters measured in EM compared to both the constant curvature and constant area models (Figs. 3h and 5i). These findings strongly support a model where flat-to-curved transition correlates with the concomitant change in the AP2/clathrin ratio.

### Membrane tension influences the flat-to-curved transition

**Fig. 6:**
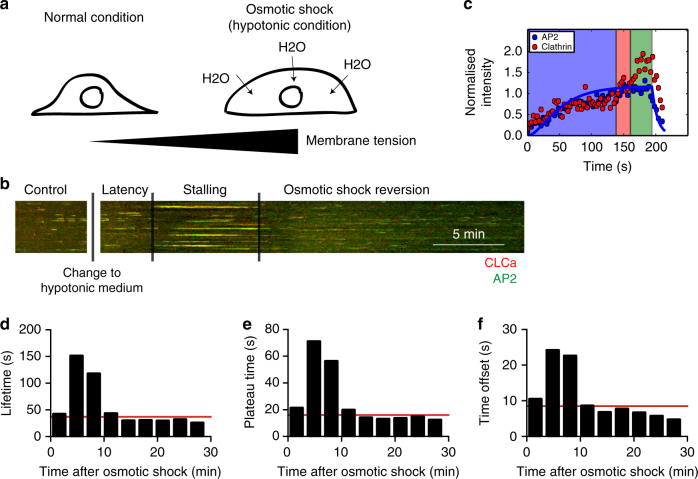
Osmotic shock induces stalling of CCSs. **a** Illustration of the effect of osmotic shock on BSC-1 cells. Hypotonic medium was applied to BSC-1 cells, inducing osmotic swelling that results in an increase in PMT. The same BSC-1-expressing fluorescently tagged clathrin light chain and AP2 proteins were followed from 5 min prior (internal control) until 30 min post hypotonic medium application using spinning disc confocal microscopy. **b** Kymograph of AP2-eGFP (green) and clathrin light chain a-tdtomato (red)-expressing BSC-1 cells. The dynamics of CCSs was recorded during 5 min prior to osmotic shock until 30 min post osmotic shock. The time after applying the hypotonic medium can be divided into latency, stalling and osmotic shock reversion time depending on the effect on CME dynamics. Scale bar: 5 min. **c** Representative AP2 (blue) and clathrin (red) intensity profile from an individual CME event during the time of stalling fitted to Eq. 1 to quantify the plateau time. **d** Quantification of the lifetime of CME events during osmotic shock experiments for 1607 tracks of one single cell. CME events were binned in 3 min intervals in respect to the onset of osmotic shock. Red line indicates lifetime of CME prior to osmotic shock. **e** Quantification of the plateau time of AP2 of individual CME events during osmotic shock experiments (as defined in Fig. 5a) for 1607 tracks of one single cell. CME events were binned in 3 min intervals in respect to the onset of osmotic shock. Red line indicates plateau time of CME prior to osmotic shock. **f** Quantification of the time offset between AP2 plateau and clathrin maximum of individual CME events during osmotic shock experiments (as defined in Fig. 5a) for 1607 tracks of one single cell. CME events were binned in 3 min intervals in respect to the onset of osmotic shock. Red line indicates time offset of CME prior to osmotic shock

By inducing curvature to the PM, the CCS needs to act against the plasma membrane tension (PMT). Higher PMT has been shown to increase the lifetime of clathrin events at the PM^28,29^ and modelling of the energetic cost of membrane bending suggests that it affects the morphology of the CCSs^30,31^. However, the effects of increasing PMT on the ultrastructural organisation of CCSs have not been investigated in living cells. We monitored the dynamics of clathrin and AP2 during osmotic shock in which the PMT was increased by applying hypotonic medium inducing osmotic swelling of the cells^28^ (Fig. 6a). Following a short latency period, we observed that CCSs stalled at the PM. This effect was transient and cells quickly reverted to normal clathrin dynamics (Fig. 6b, d). Since the growth dynamics of clathrin is stalled under osmotic conditions, we now determined the flat-to-curved transition time not by determining the time when AP2 reaches is plateau, but by calculating when CLC intensity exceeds the AP2 intensity by 5%, which we controlled to yield similar results under unperturbed isoosmotic conditions (Supplementary Information). Using fitted AP2 and unfitted CLC intensity profiles under osmotic shock, we showed that the CCSs display a longer AP2 plateau phase (Fig. 6c, e) and that the time offset was increased compared to mock treated cells (Figs. 6f and 5b). According to our findings that the change in AP2/clathrin ratio coordinates the flat-to-curved transition of CCSs, the delayed offset in the AP2/clathrin ratio under higher PMT suggests that the flat-to-curved transition is suppressed and that the coats are flat under this condition.

**Fig. 7:**
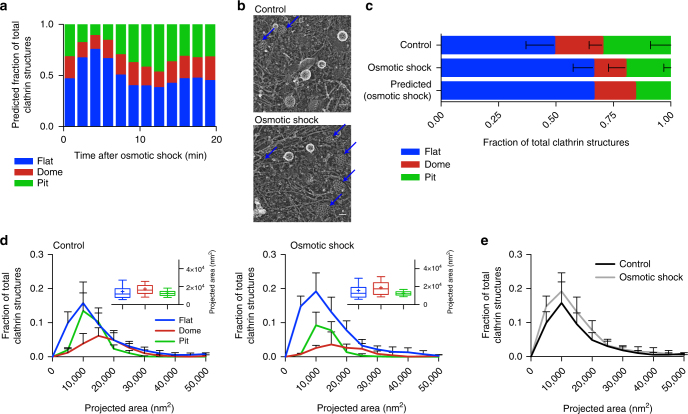
Osmotic shock blocks flat-to-curved transition of CCSs. **a** Predicted ratio of flat (blue), dome (red) and pit (green) structures calculated from the binned AP2 and clathrin profiles of CME events (Fig. 6c) during osmotic shock for 1357 tracks. **b** Examples of CCSs under normal and osmotic shock conditions. Blue arrows point to flat structures. **c** Comparison of measured and predicted frequency of flat, dome and pit structures under normal and osmotic shock conditions. **d** Projected area distribution of the different clathrin morphologies under normal and osmotic shock conditions. A box /whisker plot of the projected area is shown in the inset. Mid-line represents median, cross represents the mean and the whiskers represent the 10 and 90 percentiles. **e** Comparison of projected area distributions of flat CCSs under normal and osmotic shock conditions. Results are calculated from four different membranes (number of CCSs per membrane: normal conditions 267, 308, 229 and 323; osmotic shock: 395, 99, 351 and 201); means with SD are shown

By looking at the AP2/clathrin ratio during osmotic shock, we predicted that during the stalling phase, 70% of the CCSs would be flat (Fig. 7a). To test this notion, we performed EM of metal replica of CCSs under osmotic shock (Fig. 7b). We found an accumulation of flat CCSs under osmotic shock compared to normal conditions and the frequency was comparable to our predictions from the AP2 and CLC profiles (Fig. 7c). These flat structures, as well as the dome and pit structures, have the same size distribution as under normal conditions (Fig. 7d box/whiskers and 7e). The EM data confirm that under higher PMT the flat-to-curved transition of CCSs is inhibited. Using our data analysis pipeline from above and individual clathrin and AP2 intensity profiles acquired under osmotic shock, we could predict the morphology of the stalled CCSs. These findings are in agreement with our conclusion from above that the change in clathrin/AP2 ratio represents the precise moment at which the flat-to-curved transition occurs.

## Discussion

The complex coordination of CCS formation during CME has been investigated for decades^32,33^ and the field has been driven by the competition between the constant curvature versus the constant area models^13^. Recent technological advances, in particular in CLEM, favoured the constant area model^19^. In this work, we implemented a multidisciplinary approach to combine information from EM, fluorescence intensity profiles of individual endocytic events and mathematical modelling of CCS growth to integrate different data sets into one common framework for the analysis of the ultrastructural rearrangement of CCSs during CME.

**Fig. 8:**
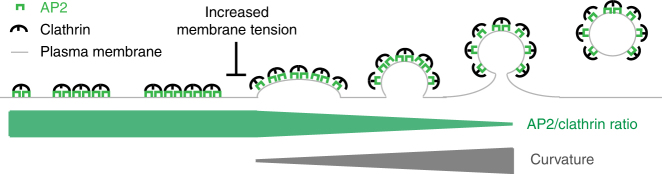
Model of CCP assembly. Schematic representation of the growth model of CCSs. CCSs initiate as flat clathrin array. They first grow in size in a flat morphology with a constant AP2/clathrin ratio. When they reach around 70% of their full clathrin content, the AP2/clathrin ratio starts to decrease and the CCSs start acquiring their curvature. CCPs keep growing by adding additional clathrin molecules until formation and release of CCVs into the cytoplasm. The flat-to-curved transition of CCSs can be inhibited by increasing PMT, resulting in an accumulation of flat structures. We propose that flat-to-curved transition is concomitant with bypassing the energy barrier necessary to curve the PM and that this critical step in CME is coordinated by the uncoupling of clathrin and AP2 characterised by their abrupt ratio decrease

By modelling the growth behaviour according to the two proposed growth models, we could calculate the expected size and morphology distribution of CCSs (Figs. 1 and 3). We could clearly demonstrate that neither of the proposed models explain the ultrastructural organisation and size distribution of CCSs present in BSC-1 cells. Instead, our data support a model in which CCSs first grow flat and then the flat-to-curved transition occurs at around 70% clathrin content (Fig. 8). Importantly, we demonstrated that this transition is directly linked to PMT and correlates with a change of the AP2/clathrin ratio within the coat. Increasing PMT results in inhibition of the change in AP2/clathrin ratio and the subsequent stalling of the ultrastructural rearrangement. Our conclusion that a change in AP2/clathrin ratio drives the flat-to-curved transition is consistent with our recent observation that AP2 (and other adaptor/accessory proteins) partitions in different nanoscale areas of the clathrin coat and that the concentration of AP2 varies within these zones at various stages of CCS assembly^22^.

Our conclusions on CCP assembly, determined in this work in BSC-1 cells, may also apply for other cell types. Because our model assumptions are minimal, we expect them to describe the most generic aspects of CME. To test whether our growth model could explain the ultrastructural distribution of CCSs described in previous studies, we extended our mathematical model and calculated the predicted contact angle between the clathrin cage and the PM, the coated surface area and the radius of tip curvature of the CCS (Supplementary Fig. 3). By comparing the model to the Avinoam et al.^19^ data set describing the distribution and ultrastructural organisation of CCSs in SK-MEL-2 cells, we could demonstrate that our growth model could also explain their observed distribution.

It was proposed earlier that the physical properties of the PM are influencing the morphology of CCSs because PMT energetically acts against curvature acquisition of the clathrin array^28,30,31^. We demonstrated the effect of increased PMT on CCS assembly in cells. Under high PMT, the accumulation of stalled flat CCSs at a stage prior to the change in AP2/clathrin ratio reveals an important step of CCP formation to overcome the PMT to acquire curvature. It is tempting to speculate that this change of AP2/clathrin ratio is a key event mandatory for curvature acquisition. The demonstrated interplay between AP2 and PMT shows that both biochemical and physical factors regulate CME. Surprisingly, the increase in PMT only changes the ratio between the different morphologies of CCSs in favour of flat structures but does not affect their size (Fig. 7d, e). One could assume that a higher energy barrier of the PM might be counteracted by the clathrin system by the formation of larger flat CCSs, which could accumulate more energy for the bending process by molecular crowding of clathrin itself as well as BAR proteins incorporated in the coat^30,34–36^. Since under high PMT the flat CCSs still have the same size as under normal PMT, we suggest that there is an internal limitation of the coat size that might be regulated by certain components of the coat. Proteins that regulate the size of CCV have been reported^37,38^ and it might be possible that the size of a flat clathrin lattice is controlled in a similar way. The fact that other cell types show much larger flat CCSs under normal conditions, commonly referred to as clathrin-coated plaques^10,15^, illustrates that the clathrin machinery is capable of forming large flat structures under certain conditions. However, the factors necessary for clathrin-coated plaque formation have not been described so far. These clathrin-coated plaques might contribute to CME. Live-cell FM^39,40^ and EM^41^ of such structures support budding of CCV from such plaques most probably from the edge, again illustrating the ability of a flat CCS to rearrange into a CCP, further supporting that our observed flat-to-curved transition is indeed possible.

We could show that during coat assembly the AP2/clathrin ratio changes. This finding is in agreement with other FM^26,27,29^ and CLEM^22^ studies. Combining mathematical reasoning and CLEM demonstrated the correlation between the change in the AP2/clathrin ratio and the time of coat bending. We found that the biggest flat structures contain the same amount of AP2 compared to fully formed CCPs, while the clathrin content still increases an additional 30% as the coat rearranges from flat to CCPs. This suggests that acquisition of curvature is not linked to addition of additional AP2 in the coat and strongly suggests that other proteins, in conjunction with clathrin, might be involved in driving the ultrastructural rearrangement of the coat. Several other adaptor and accessory proteins have been proposed to influence the ultrastructure of the CCS. Depletion of FCHO 1 and 2 has been reported to alter the ordered hexagonal organisation of the flat clathrin lattices^42^. Other proteins like CALM and NECAP have been proposed to regulate the final size of a CCV^37,38^.

Our approach provides a unifying view on the process of CCP assembly where we demonstrate that CCPs initially grow as flat lattices and that change in clathrin/adaptor ratio correlates with the onset of coat curvature acquisition prior to the completion of coat polymerisation. We propose that the proportion of different coat proteins could ultimately define the morphology of a clathrin structure and temporal changes of this proportion might initiate bending of the coat, allowing for dynamical regulation by the cell.

## Methods

### Cell lines and cell culture

BSC-1 cells were obtained from ATCC. BSC-1 cells stably expressing AP2-eGFP were created by transfecting BSC-1 cells with a plasmid expressing the sigma2 subunit of AP2 fused to eGFP^20^. Following selection with G418 (750 µg ml^−1^) (Gibco), AP2-eGFP-expressing BSC-1 cells were grown in Dulbecco's modified Eagle's medium (Gibco) supplemented with 10% foetal bovine serum (FBS), penicillin and streptomycin (Gibco) at 37 °C and 5% CO_2_. For passaging, cells were rinsed with phosphate-buffered saline (PBS) and incubated for 3–5 min with 0.05% Trypsin/EDTA (Gibco) at 37 °C and 5% CO_2_. After detaching, the cells were resuspended in complete medium. Passaging was done every 2–3 days in a ratio of 1:5–1:10.

### Transfection

Transfection of cells was done using Lipofectamine 2000 (Invitrogen). Cells were plated in 6-well plates 1 day before transfection. The next day, cells were transfected at 70–80% confluence. Then, 2 µg DNA and 8 µl Lipofectamine 2000 were separately mixed with 100 µl OptiMEM (Gibco). The two solutions were mixed together. After incubation for 20 min at room temperature, the transfection mix was added drop-wise onto the cells. For generation of stable cell lines, the growth medium was exchanged for fresh growth medium after 8 h. The cells were put under selection 2 days after transfection.

For live-cell imaging of BSC-1, AP2-eGFP transiently expressing CLCa-tdtomato were seeded 8 h after transfection.

### Antibodies and plasmids

Anti-clathrin heavy chain antibody (X22, ab2731, 1:500 for immunofluorescence) was purchased from Abcam. Anti-clathrin light chain antibody (Con.1, C1985, 1:500 for immunofluorescence) was purchased from Sigma-Aldrich. Secondary AlexaFluor 647 goat anti-mouse (A21235, 1:1000 for immunofluorescence) was purchased from Molecular Probes and goat anti-mouse Atto594 (76085-1ML-F, 1:200 for immunofluorescence) was purchased from Sigma-Aldrich. Wheat Germ Agglutinin AlexaFluor 647 conjugate (W32466, 1:200 for immunofluorescence) was purchased from Molecular Probes.

Mammalian expression vectors containing rat CLCa N-terminally fused to tdtomato^43^ and the rat AP2 subunit sigma2 C-terminally fused to eGFP^20^ were used for stable and transient expression of fluorescently tagged proteins.

### Live-cell microscopy

Glass coverslips (TH. Geyer, 25 mm diameter, No. 1.5H) were coated with poly-d-lysine solution (Sigma-Aldrich, #P6407) at a concentration of 0.1 mg ml^−1^ for 5 min at room temperature and washed three times with PBS. Cells were seeded on poly-d-lysine-coated coverslips and live-cell microscopy was performed 12–16 h after seeding. Live-cell imaging of AP2-eGFP was performed with an inverted spinning-disk confocal microscope (PerkinElmer), with a 60× (1.42 numerical aperture, Apo TIRF, Nikon) or 100× (1.4 numerical aperture, Plan Apo VC, Nikon) oil immersion objective and a CMOS camera (Hamamatsu Ocra Flash 4). An environment control chamber was attached to the microscope to keep 37 °C and 5% CO_2_. The 10-min-long movies of representative cells were taken with one frame every 3 s. Live-cell imaging of AP2-eGFP together with CLCa-tdtomato was performed with an inverted Ti microscope (Nikon) with objective TIRF illumination, with a 60× (1.49 numerical aperture, Apo TIRF, Nikon) oil immersion objective and EMCCD camera (Andor iXon Ultra DU-897U). An on-stage incubation chamber was used to keep 37 °C and 5% CO_2_. The 10-min-long movies of representative cells were taken with one frame every 3 s.

### Osmotic shock experiments

For live-cell microscopy of cells under osmotic shock, one cell was imaged for 5 min under normal conditions. Afterwards the medium was changed to hypotonic medium (1:1 ratio of medium to water with 10% FBS) and the same cell was imaged for an additional 30 min. For TEM of cells under osmotic shock, cells were put into hypotonic medium and unroofed after 10 min of osmotic shock. For the unroofing, a 1:1 ratio of stabilisation buffer to water was used. The samples were then prepared for TEM.

### Unroofing

Cells were seeded on poly-d-lysine-coated coverslips (25 mm). Unroofing was performed 16 h after seeding. Cells were washed three times with stabilisation buffer (30 mM HEPES buffer, brought to pH 7.4 with KOH, 70 mM KCl, 5 mM MgCl_2_). Unroofing was performed in 2% paraformaldehyde in stabilisation buffer using two short sonication pulses. Sample was immediately put into fresh 2% paraformaldehyde solution and fixed for 20 min at room temperature.

### Immunofluorescence

For immunofluorescence, intact cells growing on poly-d-lysine-coated 12 mm coverslips (#1.5, Thermo Scientific) or unroofed PMs were fixed with 2–4% paraformaldehyde for 20 min at room temperature. Intact cell were permeabilised with 0.5% Triton X in PBS for 15 min. After blocking with PBS and 1% bovine serum albumin (BSA) for 1 h at room temperature, samples were incubated with primary antibody diluted in PBS with 1% BSA for 1 h at room temperature. After five washes with PBS, samples were incubated with the secondary antibody. Wheat germ agglutinin diluted in 1% BSA in PBS was incubated on cells for 30 min at room temperature. After five washes with PBS, samples for STED were mounted using Mowiol and samples for correlative light and electron microscopy were fixed by incubation with 2% paraformaldehyde for 20 min at room temperature and washed three times with PBS.

### Imaging of unroofed PMs for CLEM

Widefield fluorescent images of unroofed PMs were taken with a Nikon N-STORM microscope with a 100× oil immersion objective and an EMCCD camera (Andor Ixon Ultra DU-897). To cover an area of 1 mm^2^ a montage of 15 × 15 images with an overlap of 15% for stitching was taken. The imaged area was marked with a circle (4 mm in diameter) around the centre of the imaged area using an objective diamond scriber. The immersion oil was carefully removed from the bottom of the glass coverslip and the sample was prepared for EM.

### TEM of metal replica

Coverslips with unroofed membranes were fixed with 2% glutaraldehyde in PBS overnight. Samples were incubated with 0.1% tannic acid for 20 min at room temperature. After four washes with water, the samples were incubated with 0.1% uranyl acetate for 20 min at room temperature. After 2 washes with water, samples were dehydrated with a series of ethanol solutions (15–100%). Samples were placed in each ethanol solution for 5 min. After replacing the 100% ethanol solution, the samples were dried in a critical point dryer. The samples were then put under vacuum until they were coated. The samples were coated with JFDV JOEL Freeze Fracture Equipment with a first layer of platinum with an angle of 17° while rotating and with a second layer of carbon with an angle of 90° while rotating. For better orientation, the marked area of the coated samples was imaged with a phase contrast microscope. The samples were then cut to fit on the EM grids (TED PELLA, 75 Mesh Copper, Support Films Formvar/Carbon). Then, 5% hydrofluoric acid was used to remove glass from the metal replica. The floating metal replica was extensively washed with water and then carefully placed on a glow discharged EM grid. Samples were dried on filter paper and again imaged with a phase contrast microscope. TEM imaging was performed using a JEOL 1400 equipped with SerialEM freeware for montaging. Montages of large membrane sheets at 12,000 magnification (1.82 nm per pixel) with 10% overlap were imaged.

### Transformation of images for CLEM

The FM image and the EM montage of the same membrane sheet were first manually and roughly overlaid using Photoshop. MATLAB was used to transform the fluorescence image according to the  EM montage using three manually identified CCSs. For the transformation, the centre of the clathrin structure in the  EM montage and the centre of the fluorescence signal determined by a Gaussian fit were used as landmarks.

### TEM and CLEM analysis of CCSs

For the TEM analysis, CCSs were manually identified. The projected area was measured by outlining their surrounding using ImageJ/Fiji. Classification into flat, dome and pit structures was done by visual evaluation of their curvature. Flat structure can be distinguished from curved structures. Within the curved coats, pit structures were discriminated from dome structures by the existence of a highly contrasted surrounding ring, which represents a constricted neck. For the CLEM analysis, the fluorescent intensity was correlated to a CCS identified in TEM. For fluorescence intensity measurements, background correction was performed by rolling ball algorithm using ImageJ/Fiji. Objects were manually defined and the sum of the fluorescence intensity of all pixels over the object was measured on the transformed FM image.

### Tracking

For tracking CME events, we used ilastik (http://ilastik.org). First, the images were segmented using the pixel classification and object classification workflow. For tracking, the automatic tracking workflow was used. The maximal distance was put to 5 to avoid merging of close tracks. Background correction was performed by rolling ball algorithm using ImageJ/Fiji. For fluorescence intensity measurements of tracked objects, the sum of the fluorescence intensity of all pixels over the object was measured. Automatic tracking of CCSs using AP2-eGFP-expressing BSC-1 cells was performed using a probabilistic particle tracking method^44^. The method combines Kalman filters with particle filters and probabilistic data association with elliptical sampling (PDAE). For particle detection, a Laplacian-of-Gaussian filter and connected-component labelling was used. Based on the computed trajectories, the signal intensity of each tracked object (normalised to the background intensity) was determined and intensity statistics over all trajectories were computed. Also, the object size was determined for each time. In addition, the lifetime of CCSs was quantified and classified into different ranges.

### STED

STED nanoscopy was carried out using the Two-colour-STED system (Abberior Instruments GmbH, Göttingen). Image acquisition was performed using a 100× Olympus UPlanSApo (NA 1.4) oil immersion objective and 70% nominal STED laser power (*λ* = 775 nm, max. power = 1.2 W). Line accumulation was set to 4 and a pixel size of 15 nm was used. Deconvolution of acquired images was done using imspector software (Abberior Instruments GmbH, Göttingen). Richardson-Lucy deconvolution with a regularisation parameter of 0.001 was used and stopped after 30 iterations.

### Mathematical growth laws

Details on the mathematical approach used to calculate the distribution of the various CCSs according to the various growth models are available in Supplementary Information.

### Code availability

The code used to generate the findings of this study is available upon request from Ulrich S. Schwarz (schwarz@thphys.uni-heidelberg.de).

### Data availability

The EM, CLEM and FM data sets generated and analysed in this study are available at 10.6084/m9.figshare.5802903.

## Electronic supplementary material

Supplementary Information(PDF 949 kb)

Peer Review File(DOCX 774 kb)
