# Supplementary material for: Clathrin-adaptor ratio and membrane tension regulate the flat-to-curved transition of the clathrin coat during endocytosis
Source: Nat Commun. 2018 Mar 16;9:1109. doi: 10.1038/s41467-018-03533-0 (PMC5856840; doi:10.1038/s41467-018-03533-0)
Supplement: Supplementary file 1 — Supplementary Information(PDF 949 kb) [file 41467_2018_3533_MOESM1_ESM.pdf]

Clathrin-adaptor ratio and membrane tension regulate the flat-to-curved transition of the clathrin coat during endocytosis

Bucher et al. NCOMMS-17-26069-T

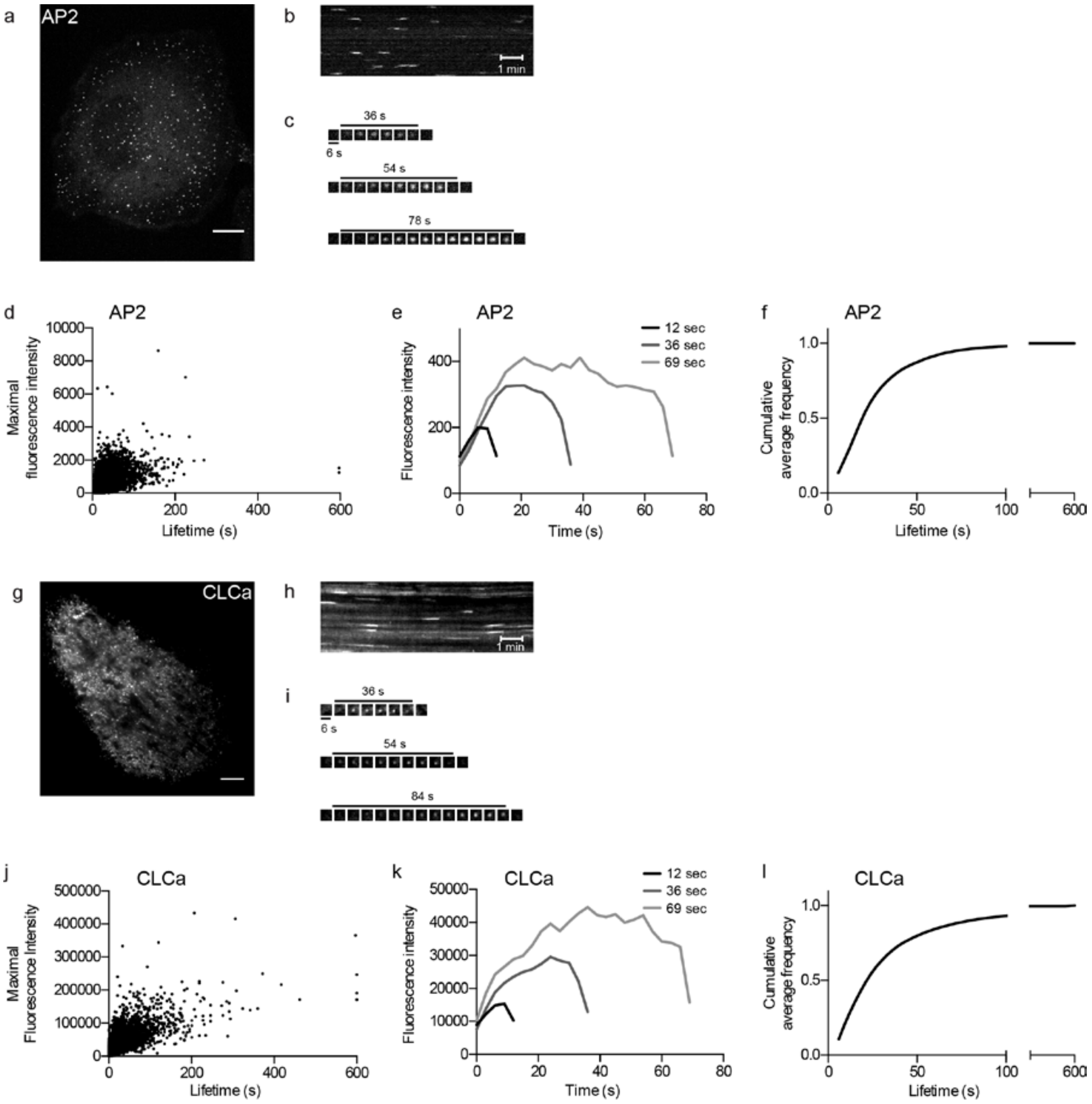

Supplementary Figure 1: Characterisation of clathrin dynamics in BSC-1 cells. Spinning disc live-cell microscopy of BSC-1 AP2-eGFP cells. (a) Representative image of a BSC-1 AP2-eGFP cell. Scale bar: 10μm. (b) Kymograph of a 10 minute long movie following AP2-eGFP signal. Scale bar: 1min (c) Representative individual CME events. (d) Dot plot showing the lifetime and the maximal fluorescence intensity of AP2-eGFP of all CME events from one cell in a 10 minute long movie. Total number of tracked CME events: 14,694. (e) Average fluorescence intensity profiles (AP2-eGFP) of CME events with lifetime of 12, 36, and 69 seconds. Data are acquired from one cell during a 10 minute long movie. Number of tracks used for calculating the average intensity profiles: 1,525 for 12sec-lifetime; 604 for 36sec-lifetime; 230 for 69sec-lifetime. (f) Cumulative average frequency of CME lifetime. The average was calculated from five different cells. Number of total tracks per cell: 14,037; 16,386; 14,694; 16,797; 19,631. (g) TIRF live-

cell microscopy of BSC-1 cells expressing clathrin light chain a (CLCa)-tdtomato. Representative image of a BSC-1 cell expressing CLCa-tdtomato. Scale bar: 10μm. (h) Kymograph of a 10 minute long movie following CLCa-tdtomato. Scale bar: 1min (i) Representative individual CME events. (j) Dot plot showing the lifetime and the maximal fluorescence intensity of CLCa-tdtomato of all CME events from one cell in a 10 minute long movie. Total number of tracked CME events: 4,763. (k) Average fluorescence intensity profiles (CLCa-tdtomato) of CME events with lifetime of 12, 36, and 69 seconds. Data are acquired from one cell during a 10 minute long movie. Number of tracks used for calculating the average intensity profiles: 175 for 12sec-lifetime; 75 for 36sec-lifetime; 21 for 69sec-lifetime. (l) Cumulative average frequency of CME lifetime. The average was calculated from four different cells. Number of total tracks per cell: 2,429; 4,763; 5,443; 16,797; 8,393.

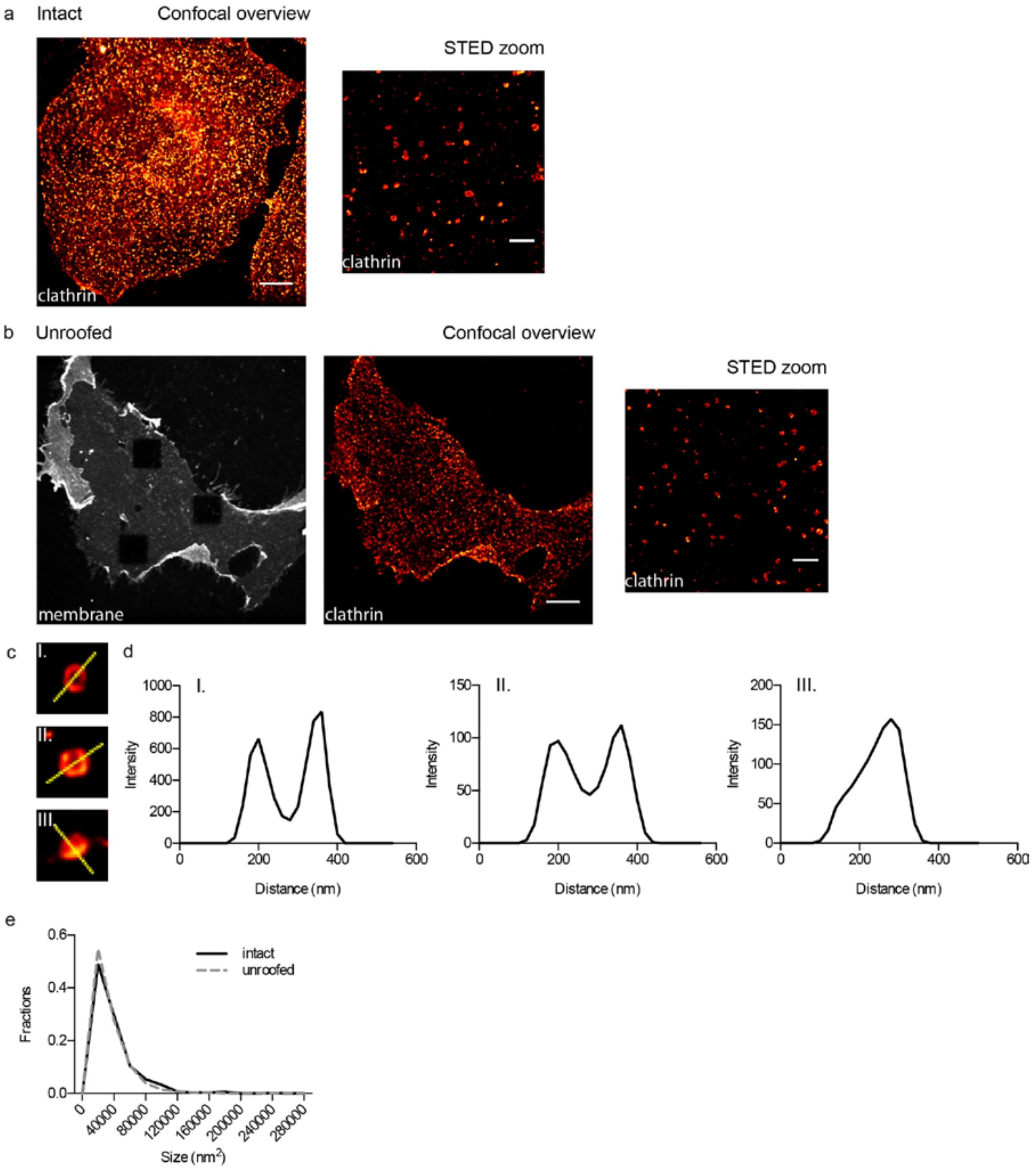

Supplementary Figure 2: Comparative STED nanoscopy analysis of CCS in intact *vs.* unroofed cells. (a) STED nanoscopy of intact BSC-1 cells. CCS were immunostained with an anti-clathrin light chain (CLC) antibody. Left: confocal overview, scale bar: 5µm. Right: STED view, scale bar 1µm. (b) STED nanoscopy of unroofed BSC-1 cells. BSC-1 cells were unroofed by sonication; the remaining attached PM was stained with wheat germ agglutinin (WGA). CCS were immunostained with an anti-clathrin antibody. Left: confocal overview, scale bar: 5µm. Right: STED view, scale bar 1µm. (c-d) Example of different CCS and their intensity line profile. Yellow line marks the axes measured for the intensity line profiles. (e) Analysis of the size distribution of CCS in intact and unroofed cells. CCS from five cells per conditions with three STED pictures per cell.

Number of analysed CCS for intact cells: 820 and unroofed cells: 1169.

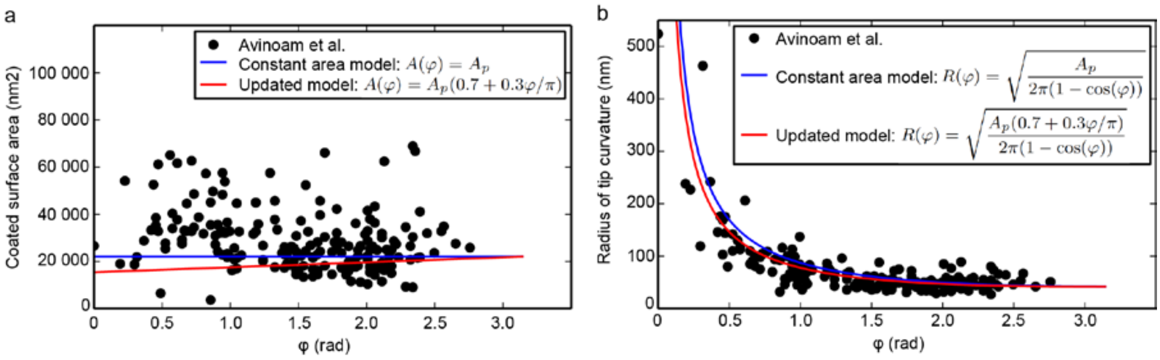

Supplementary Figure 3: Updated growth model explains the CLEM data from Avinoam *et al.* (a) Clathrin coated surface area as a function of the growth angle φ. The constant area model where we assume A=22000nm^2, corresponding to the most probable surface area[1] is plotted (blue). Our updated model where we assume that the surface starts to bend when 70% of the final clathrin content is reached and grows the last 30% linear with φ plotted (red). The data (black dots) were extracted from Avinoam *et al.*[2]. (b) Radius of tip curvature as a function of the growth angle φ. Again, the constant area model (blue) and our updated model (red) are plotted. The radius of the tip curvature is given assuming that the clathrin structure exhibits the shape of a spherical cap. Then the tip curvature reads $R = \sqrt{A(\theta)/(2\pi(1-\cos(\theta)))}$. The data (black dots) were extracted from Avinoam *et al.* [2].

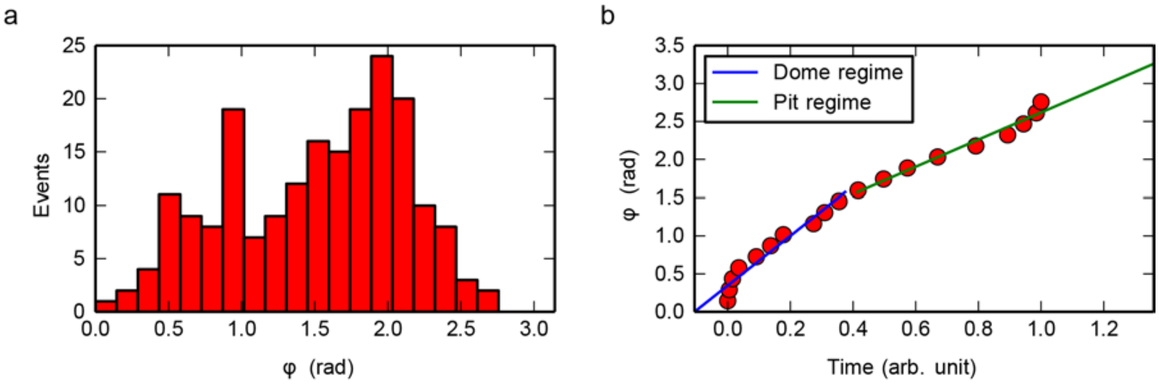

Supplementary Figure 4: Dome and pit transformation time deduced from Avinoam *et al.* (a) Histogram of the growth angle φ (red) extracted from Avinoam *et al.*[2] (b) The growth angle φ as a function of the normalised sum of all structures with growth angle smaller or equal to φ (red) which is proportional to a time assuming a linear time relationship between φ and time. From the linear fit to the data with φ smaller than $\pi/2$ for domes (blue) and with φ larger than $\pi/2$ for pits (green) we compute the ratio of transformation times for domes and pits and obtain roughly 2:3.

Supplementary Methods

Constant area model

As the fluorescence intensity of labelled clathrin triskelia is proportional to the number of incorporated clathrin triskelia or equivalently to the size of clathrin covered membrane area, we model the assembly of CCV as surface growth.

Our mathematical description of the constant area model considers growth of a flat circular patch with radius r only at its edge (L) with rate k_{on}. To ensure limited growth there has to be some balancing process to prevent a runaway-process. Mathematically the simplest idea is that growth is regulated by the size of the domain area (A). Effectively we model this as dissociation over the area of the patch with rate k_{off} , which can be expressed by $\frac{dA}{dt} = k_{\text{on}}L - k_{\text{off}}A$. In (Fig. 3a) a sketch of the growth schematics is shown. The growth equation can be simplified by plugging in the surface of a circular patch $A = \pi r^2$, it follows $\frac{dr}{dt} = k_{\text{on}} - k_{\text{off}}\frac{r}{2}$ with steady state radius $r_{\text{ss}} = 2k_{\text{on}}/k_{\text{off}}$. By integrating this equation, we find the patch area as a function of time

$$A(t) = \pi\left(\frac{2k_{\text{on}}}{k_{\text{off}}}\left(1 - \mathrm{e}^{-(\mathrm{k_{off}}/2)\mathrm{t}}\right)\right)^2.$$

In the observed fluorescence intensity tracks the intensity decreases after some time until the intensity vanishes completely. Biologically, this indicates that the clathrin coated vesicle pinches off the cell membrane and therefore, moves out of the focus of the microscope. We model this by assuming an exponential decay of the area with time constant τ, starting at time t_{decrease}

$$A(t) = \begin{cases} \pi\left(\frac{2k_{\mathrm{on}}}{k_{\mathrm{off}}}\left(1-\mathrm{e}^{-(\mathrm{k_{off}}/2)\mathrm{t}}\right)\right)^2 & t \le t_{\mathrm{decrease}}, \\ \pi\left(\frac{2k_{\mathrm{on}}}{k_{\mathrm{off}}}\left(1-\mathrm{e}^{-(\mathrm{k_{off}}/2)\mathrm{t}}\right)\right)^2 \mathrm{e}^{-\mathrm{t}/\tau} & t > t_{\mathrm{decrease}}. \end{cases} \quad \text{(Eq. S1)}$$

We note that modelling the pinching as an exponential decay only serves as a measure to robustly define the end-point of a fluorescence track. No further information is extracted from this.

As the steady state is only reached approximately we define an area plateau at 95% of the steady state area and the corresponding time t_{plateau}. The constant area model assumes that a flat patch transforms into a spherical pit as soon as the patch reaches the area plateau. Here, we neglect the exact details of the transformation process but classify the CCS as flat, dome (less than a half sphere) or pit according to time. Before reaching the plateau, the area is considered flat. After reaching the plateau we classify CCS in the first 40% of the remaining time until t_{decrease} to be domes and in the last 60% of time to be pits. For the exact choice we use our EM data (see main text) and the data set of Avinoam *et al.*[2] and calculate the histogram $p(\varphi)$ of the growth angle φ. The number of structures which one finds between φ and $\varphi + \mathrm{d}\varphi$ should be proportional to the time it takes for a structure to evolve from φ to $\varphi + \mathrm{d}\varphi$, assuming a linear time course. Therefore, φ as a function of the integral should give a measure of the time course. Since we are mostly interested in the number of domes and pits, we approximate the time course of φ for domes and pits by assuming a different linear relationship between φ and time. By dividing the time which it takes to form a half sphere by the time it takes to from a full sphere we end up with a forming ratio time of domes and pits of roughly 2:3 (Supplementary Fig.4).

Data fits

To test whether the constant area model correctly describes the shape and size of clathrin coated vesicles we fitted Eq. 1 to 4927 FM tracks of 4 different cells (Fig. 3b) and calculated from the fitted area surface growth curves histograms which we could compare to EM histograms. Therefore, we related the intensity of an FM track to its

corresponding area. Furthermore, the FM dataset was filtered before the fitting. The exact details of our procedure are described in the following.

Relate fluorescence intensity and surface area by means of CLEM

To relate the fluorescence intensity of a clathrin FM track to the corresponding clathrin covered membrane area we use our clathrin CLEM data, relating the projected surface size of CCS to their fluorescence intensity. We analyse flat CCS for which the projected area directly corresponds to their surface. By fitting a line through the origin to the clathrin CLEM data we get the slope β providing us with a linear relation between size and intensity. As the local intensity background is removed from the CLEM data we find

$I(A) = \beta A$, (Eq. S2)

where I is the intensity of the FM data, A is the area of the clathrin structure and β is the proportionality constant. Fig. 4b shows the intensity of flat structures as a function of their projected area (blue). We find $\beta = 4085 nm^2$.

Relate different FM datasets

To calculate a size histogram from fluorescence intensity tracks we analyse live cell FM data, that have a different intensity level than the fluorescence intensity of the CLEM data. Therefore, we need to relate these two different data sets. As the CLEM intensity I and the live cell FM intensity I' are both proportional to the number of labelled clathrin triskelia, both intensities, which are background corrected, can be related only by some factor α

$I' = \alpha I \rightarrow \alpha = \langle I' \rangle / \langle I \rangle$, (Eq. S3)

which can be calculated by dividing the means (indicated by $\langle\rangle$) of both data sets.

Relate EM and FM datasets

We next restricted the live cell FM data set to ensure that the calculated size histogram is comparable to the EM histogram. In EM we only detect structures that reach a threshold size. However, in the live cell FM data set we register only detectable intensities, which exceed the local background signal. Therefore, we relate the minimal

detectable size in CLEM (A_{T}) to a threshold intensity (I_{T}), which we relate to a threshold intensity ($I_{\mathrm{T}}^{'}$) that we use on the live cell FM tracks. As $I^{'} = \alpha I$ we find

$$\frac{\langle I^{'} \rangle - \langle I_{\mathrm{T}}^{'} \rangle}{\langle I^{'} \rangle} = \frac{\langle I \rangle - \langle I_{\mathrm{T}} \rangle}{\langle I \rangle}$$

As $\langle I^{'} \rangle$ is a function of $\langle I_{\mathrm{T}}^{'} \rangle$ we can find the root of

$$0 = I_{\mathrm{T}} \frac{\langle I^{'} \rangle}{\langle I \rangle} - I_{\mathrm{T}}^{'}$$

by iteratively increasing $\langle I_{\mathrm{T}}^{'} \rangle$.

In EM we find that the minimal sized clathrin structure has an area of $A_{\mathrm{T}} = 5644 nm^2$, corresponding to a calculated intensity of $I_{\mathrm{T}} = 2.305 \cdot 10^7 \mathrm{arb.\,unit}$. We find the mean intensity in the clathrin CLEM data of all structures $\langle I \rangle = 1.766 \cdot 10^8 \mathrm{arb.\,unit}$. To measure the mean intensity in the live cell FM data set we sample from each FM track that lasts a least 24 seconds a number of intensities proportional to its lifetime. From these sampled intensities, which we restrict to be larger than the threshold value $I_{\mathrm{T}}^{'}$, we then compute the mean intensity. We obtain $I_{\mathrm{T}}^{'} = 5.450 \cdot 10^3 \mathrm{arb.\,unit}$, $\langle I^{'} \rangle = 4.148 \cdot 10^4 \mathrm{arb.\,unit}$. By combining the Eq. S2 and Eq. S3 we finally arrive at

$$A^{'}(I^{'}) = \frac{I^{'}}{\alpha\beta}$$

according to which we compute the surface of a clathrin structure from its intensity in the live cell FM. Furthermore, we filter all FM tracks showing a mean intensity which is smaller than $I_{\mathrm{T}}^{'}$, as those tracks would not be observable in EM.

Data filtering

Many CCS at the plasma membrane represent abortive structures that will not commit to form a CCP. To ensure that abortive structures are not analysed in our study, dynamic tracks with a lifetime inferior to 24 seconds were excluded (data presented in this manuscript). Of note, we also repeated our analysis using a minimal lifetime of 12 seconds and found similar results (data not shown).

We filter our data set for FM tracks with multiple structures (defined as a FM track that shows at least two clear intensity maxima) to allow for direct fitting of single tracks. Precisely, we check for each data point of an intensity track if its intensity exceeds 80% of the mean intensity to find a „first plateau“. If a data point after a „first plateau“ falls below 40% of the mean intensity we find a „first low“. If a data point after the „first low“

exceeds the mean again by 20% we found a structure with multiple intensity maxima. Such tracks are filtered out. Furthermore, we filter tracks that start already with a mean intensity level $I'(t=0) > 0.5\langle I' \rangle$.

Parameter choice and data fitting

The parameters for the fit are restricted by assuming that growth curves should at least reach 90% and at most 120% of the maximal area value. Additionally, we assume that vesicle pinch off (corresponding to a decrease in the intensity to 10%) takes between 0 seconds to 20 seconds. Additionally, we require the fit to reach 99% of the steady state area before the area decrease happens and at least 10% of that time until the 99% area level are reached. We implement the Python module 'lmfit' for fitting the area tracks where we use the method 'nelder' of the minimiser function. In this way we obtain for each track three parameters with mean values (r_{ss}= 65 nm (as sphere), k_{off} = 0.5 1/s, $t_{decrease}$= 46 s) that characterise the growth curve.

Calculation of the size histogram

From the growth curves, we calculated a histogram (Fig. 3c) to compare the constant area model to the measured projected size EM histogram (Fig. 3h). We proceeded as follows:

- From each of the fitted growth curves we uniformly drew a number of time points, proportional to the time until the structure pinches off the cell membrane, that is given by $t_{decrease}$ (Fig. 3a and b). We used 4927 FM tracks of 4 different cells giving us 4 million time points, which we used to calculate the size histogram.
- We classified the chosen times and corresponding areas into three categories. If $t < t_{plateau}$ the structure is flat (blue region in Fig. 3b). We assume that the transformation process from flat to dome takes 40% of the plateau time whereas from dome to pit it takes 60% (see above). Therefore, if $t_{plateau} < t < t_{plateau} + \frac{2}{5}\left(t_{decrease} - t_{plateau}\right)$ the structure is a dome (red region in Fig. 3B) and otherwise it is classified as a pit (green region in Fig. 3b).
- We computed the projected area by assuming that the transformation within the dome and pit phase is a linear function of time. Therefore, we divided the area by $1 + \frac{5}{2}\frac{t - t_{plateau}}{t_{decrease} - t_{plateau}}$ for domes and $2 + \frac{5}{3}\frac{t - t_{plateau} - \frac{2}{5}(t_{decrease} - t_{plateau})}{t_{decrease} - t_{plateau}}2$ for pits. This

factor equals 1 for a flat patch, 2 for a completed dome (half sphere) and 4 for a completed pit (full sphere).

- We excluded times for which the corresponding area is below the area threshold, since the corresponding structures fall under the detection limit of TEM determined as being the smallest object that can be confidently identified as clathrin coat by TEM (Fig.3b, white region and dashed line) or where the area already decreases (Fig.3b, white region). This ensures that both the calculated and measured data sets are similarly restricted.
- From the calculated projected areas, we then determined the size histogram (Fig. 3c), which we compare to the EM data (Fig. 3h).

Curvature acquisition during growth: updated model

In the updated growth model we assume that CCS first grow flat, start to invaginate as they reach 70 % of their final size (which we determine by taking the inverse of the intensity ratio of pit and flat structures in clathrin CLEM, which is 1.44) and finally grow as a spherical cap until a full pit has formed. As before we model the assembly of clathrin coated vesicles as surface growth.

Our mathematical description of the "curvature acquisition during growth model " considers a spherical cap with area $A = 2\pi R^2(1 - \cos(\varphi))$ and radius R that grows again only at its edge (L) with rate k_{on} which can be expressed by $\frac{dA}{dt} = k_{\mathrm{on}}L$. Now the system has an intrinsic mechanism to stop growth, namely formation of a sphere and therefore needs no balancing mechanism anymore. In (Fig. 3d) a sketch of the growth schematics is shown. In the limit of a small growth angle $\varphi = k_{\mathrm{on}}t/R < 1$ we recover the growth equation of a flat patch $A(t) = \pi(k_{\mathrm{on}}t)^2$. For an almost complete pit the growth equation also holds perfectly. However, the mathematical description of the growth model approximates the flat patch by a spherical cap for intermediate flat patch sizes. In this case, the error is negligible as the model interpolates between the correctly addressed limiting cases and is not used to assign the shape of the CCS.

In contrast to the constant area model now no dissociation mechanism is included in the growth equation because of two reasons. First, the system has an intrinsic mechanism to stop growth and second, any area-related dissociation mechanism would imply that one

could never close the sphere completely. Therefore, to obtain simple growth law such a mechanism is neglected. We note that this choice does still allow for turnover of the clathrin coat which does not imply a net area loss.

To define the ultrastructural organisation of the clathrin lattice, we neglect, as before, the exact details of the transformation process but classify the CCS as flat, dome (less than a half sphere) or pit according to time relative to the time when reaching the maximal area. Before reaching 70% of the maximal area the coat is flat. After reaching 70% of the maximal area we classify CCS in the first 40% of the remaining time to be domes and pits otherwise (as above).

As before we model that vesicles pinch off the membrane by an exponential decay of the area with time constant τ, starting at time $t_{\mathrm{decrease}} = \pi R/k_{\mathrm{on}}$. We note that t_{decrease} carries the unit of a time since the rate k_{on} carries the unit of a velocity. The full growth equation for the area as a function of time $A(t)$ reads

$$A(t) = \begin{cases} 2\pi R^2 \left(1 - \cos\left(\frac{k_{\mathrm{on}} t}{R}\right)\right) & t \leq t_{\mathrm{decrease}}, \\ 4\pi R^2 \mathrm{e}^{-t/\tau} & t > t_{\mathrm{decrease}}. \end{cases} \quad \text{(Eq. S4)}$$

As before we note that modelling the pinching as an exponential decay only serves as a measure to robustly define the end-point of a fluorescence track.

Data fits, parameter choice and data fitting

To test whether the "curvature acquisition during growth" model correctly describes the shape and size of clathrin coated vesicles we fitted Eq. S4 to 4927 FM tracks of 4 different cells (Fig. 3e) and calculated from the fitted area surface growth curves histograms which we could compare to EM histograms. Therefore, we related the intensity of an FM track to its corresponding area. Furthermore, the FM dataset was filtered before the fitting. The exact details of our procedure are the same as before.

The parameters for the fit are restricted by assuming that growth curve should at least reach 90% and maximal 120% of the maximal area value and that the vesicle pinching off (corresponding to a decrease in the intensity to 10%) takes between 0 seconds to 20 seconds. We implement the Python module 'lmfit' for fitting the area tracks where we use the method 'nelder' of the minimiser function. In this way we

obtain for each track two parameters with mean values (R = 69 nm, t_{decrease}= 42 s) that characterise the growth curve.

Calculation of the size histogram

From the growth curves we calculated a histogram (Fig. 3f) to compare "the curvature acquisition during growth model" to the measured projected size EM histogram (Fig. 3h). We proceeded in principle as before and only mention changes:

- We classified the chosen times (around 4 million time points) and corresponding areas into three categories. If $A(t) < 0.7A_{\max}$ the structure is flat (blue region in Fig. 3e) and we call this time $t_{\text{transformation}}$. We assume that the transformation process from flat to dome takes the first 40% of the remaining time until the maximal area is reached whereas from dome to pit it takes the rest of the time (as above).
- We computed the projected area by assuming that the transformation within the dome and pit phase is a linear function of time. Therefore, we divided the area by $1 + \frac{5}{2}\frac{t - t_{\text{transformation}}}{t_{\text{decrease}} - t_{\text{transformation}}}$ for domes and $2 + \frac{5}{3}\frac{t - t_{\text{transformation}} - \frac{2}{5}(t_{\text{decrease}} - t_{\text{transformation}})}{t_{\text{transformation}} - t_{\text{plateau}}} 2$ for pits. This factor equals 1 for a flat patch, 2 for a completed dome (half sphere) and 4 for a completed pit (full sphere).

Curvature acquisition during growth: Flat-to-curved transition corroborates with the change of clathrin/AP2 ratio

Here, the only thing that changes compared to the updated model is that the transition time $t_{\text{transformation}}$ is now given by the time when the AP2 intensity plateaus, which we calculated by fitting eq. 1 to the AP2 FM intensity tracks. We use eq. 1 for the AP2 tracks as they show a clear plateau phase which is reached prior to the onset of coat curvature. Therefore, the growth of the AP2 patch is limited and eq. 1 is a simple representation of such a situation. This assumption is supported by the experimental finding that AP2 is highly dynamic throughout the coat. Indeed FRAP experiments have demonstrated the high recovery rate of AP2 with CCS (data not shown and [3]).

Live cell FM analysis resampling

To determine the ratio of clathrin and AP2 during the process of CME, we performed TIRF microscopy of BSC-1 AP2-eGFP cells transiently expressing CLCa-tdtomato. We analysed the excess of clathrin in comparison with AP2 during the formation of CCVs. Therefore, we calculated the ratio of the maximum clathrin intensity divided by the intensity of clathrin at the time when AP2 shows an intensity plateau (95% intensity level) and subtract this ratio from the ratio, which we get for AP2.

In detail: we set a threshold for the AP2 intensity underneath fluorescence intensity tracks are excluded (as before). We calculate the plateau time, defined as the time when 95% of the AP2 plateau intensity is reached by fitting the constant area model to the AP2 FM data. Additionally, we normalise all intensity values to the intensity value when 95% of the AP2 intensity (clathrin intensity) is reached. For each track (in total 754 tracks of one single cell) we calculate the difference of the time when AP2 plateaus and clathrin reach the maximum intensity and determine the corresponding histogram (cf. Fig 5b). Furthermore, we calculate the intensity offset given by the normalised maximum clathrin intensity divided by the normalised maximum AP2 intensity (cf. Fig. 5c).

Calculation of the ratio histogram during the osmotic shock

To determine the ratio histogram of flat, dome and pit CCS during the osmotic shock (Fig. 7a and 7c) we first defined a transition time $t_{\text{transformation}}$, when flat CCS start to invaginate, where the normalised clathrin intensity exceeds the normalised AP2 intensity by 5% (blue region in Fig. 6c). We assumed that the transformation process from flat to dome takes the first 40% of the remaining time until the vesicle pinches of the PM, given by t_{decrease} (red region in Fig. 6c) whereas from dome to pit it takes the remaining 60% of time (green region in Fig. 6c). Next, we started 50s after the osmotic shock and determined the morphology of all tracks present at that time depending on the description above. We repeated this procedure in steps of 5 seconds and average the number of structures over time intervals of 100 seconds. In total we used 1356 FM tracks of one cell. The found ratios of flat, dome and pit CCS were then plotted as a function of the time after the osmotic shock (Fig. 7a).

To test the consistency of this approach we used it to calculate the ratios for flat, dome and pit structures on the data without osmotic shock consisting of 4927 FM tracks

of 4 different cells. Averaging over all tracks and considering only tracks with lifetimes shorter than 90s and with AP2/clathrin discrepancy we obtained 47.8% flat CCS, 18.0% dome CCS and 34.2% pit CCS which is very similar to the determined ratios in (Fig. 5g).

Quantification of agreement between measured and predicted size histograms

To determine the level of agreement between measured (Fig. 3h) and predicted size histograms (Fig. 3c, Fig. 3f and Fig. 5e) we calculated chi-squared $\chi_j^2 = \sum_{i=1}^{k} \frac{(N_{ij}-np_{ij})^2}{np_{ij}}$ where we sum over all bins k the occurrences N_i of measured CCS and compared it with the number of expected occurrences np_i, where p_i is the predicted normalised frequency per bin, which we deduced from our models (CAM=constant are model, UM=updated model and AP2=transition flat/dome determined by the time when the AP2 intensity plateaus) and $n = \sum N_i$. We repeated this for all CCS $j = \{flat, dome, pit\}$ such that we found three values for χ_j^2 for each model and cell. We averaged these values for all CCS and four cells and found: $\chi^2_{\mathrm{CAM}} = 1018$, $\chi^2_{\mathrm{UM}} = 649$ and $\chi^2_{\mathrm{AP2}} = 632$ which shows that the model plotted in Fig. 5e describes the data best.

Additionally, we performed a Welch's t-test to calculate p-values for the null hypothesis that the measured and predicted size distributions of a CCS have identical mean values. We averaged over all CCS and four cells and found $p_{\mathrm{CAM}} = 0.60$, $p_{\mathrm{UM}} = 0.75$ and $p_{\mathrm{CAM}} = 0.78$.

Supplementary References

1. Kumar, G. & Sain, A. Shape transitions during clathrin-induced endocytosis. *Phys. Rev. E* **94,** 62404 (2016).
2. Avinoam, O., Schorb, M., Beese, C. J., Briggs, J. A. G. & Kaksonen, M. Endocytic sites mature by continuous bending and remodeling of the clathrin coat. *Science* **348,** 1369 LP-1372 (2015).
3. Wu, X. *et al.* Adaptor and Clathrin Exchange at the Plasma Membrane and trans-Golgi Network. *Mol. Biol. Cell* **14,** 516–528 (2003).
