# Supplementary material for: Clathrin-adaptor ratio and membrane tension regulate the flat-to-curved transition of the clathrin coat during endocytosis
Source: Nat Commun. 2018 Mar 16;9:1109. doi: 10.1038/s41467-018-03533-0 (PMC5856840; doi:10.1038/s41467-018-03533-0)
Supplement: Supplementary file 2 — Peer Review File(DOCX 774 kb) [file 41467_2018_3533_MOESM2_ESM.docx]

Revised version of manuscript NCOMMS-17-26069-T

Response to reviewers in chronological order

This manuscript was originally submitted to Nature cell biology and was transferred together with our answer to the reviewer to Nature communications. You will find below the NCB reviewer comments and our answers to them.

**Reviewer #1**

This is an extensive study whose results, in general, deserve publication but, in my opinion, not in a magazine of NCB level. The reason is that the study does not provide a clear-cut experimental evidence for the time course of events leading to CCP and CCV formation and, therefore, leaves open the problem of the constant area versus constant curvature mechanisms. The authors are aware of this problem and suggest that fitting a mathematical model for kinetics of CCP formation to the EM observations can replace a direct observation of the times sequence of events happening to individual clathrin assemblies. I did not find such analysis convincing.

We appreciate that reviewer 1 believes that this work represents an extensive study and deserves publication. We agree with the reviewer that we do not provide a direct experimental evidence demonstrating live the ultrastructural change of the clathrin coat during CME. However, to our knowledge such a technology is not yet available. To achieve this, one would require a technology allowing us to address live the ultrastructural organization of the coat with resolutions that are normally obtained using EM approaches (fixed samples). Although super-resolution optical microscopy (in particular lattice light-sheet microscopy) would in principle permit the visualization of live samples, this technology still does not provide enough resolution yet to look precisely at the coat ultrastructural organization (Li et al., 2015 Science). Even in studies in which complex tracking and analysis of data sets were used, detailed information on the coat organization is limited. For example, Picco et al. in eLife 2015, have aligned trajectories obtained from live fluorescence microscopy of various coat proteins and interpreted their location within the coat using information derived from CLEM. Although, with this elegant approach, they could achieve a temporal resolution of 1 s and a lateral spatial resolution of 10 nm and could measure the kinetics of selected protein localizations, they did not obtain direct dynamic details of the 3D ultrastructural organization of the coat. Similarly, Leyton-Puig et al., 2017, Nat Comm, recently reported using super-resolution microscopy that the large flat clathrin plaques are hubs of CME, suggesting that flat lattice can indeed be reshaped, but no direct visualization showing how the coat rearrangement was provided.

To advance our understanding of coat formation during CME, our strategy was to calculate the geometrical distribution of clathrin coats (size and ultrastructural organization) according to theoretical growth models (constant curvature and area models) in order to correlate two independent technical data sets (i.e. live fluorescent microscopy and EM).

In the revised manuscript, we now explain our approach in more detail which consists of mathematical modelling, EM, dynamics and CLEM data to challenge the current clathrin growth models and to propose an alternative one. Line 156-163.

The model is based on multiple assumptions, which, essentially, describe the observations, such as, for example, the onset of curvature taking 40% of the plateau time, whereas from dome to pit it takes 60%, linear time courses of transformations within the domes and pits, specific assumptions concerning the modes of association (over the rim) and dissociation (over the area) of the clathrin assemblies, the exponential course of CCP disconnection from the membrane and others. These assumptions do not seem to have behind them a clear experimental background independent of the measurements of the current work.

We agree that our modelling approach uses several assumptions as listed here, but it is important to note that each of them has a very different status. (1) The most important assumption is the one that the patch grows over the rim, which is motivated by the fact that incorporation of new triskelia in the area is non-trivial for topological and energetic reasons. To our knowledge there is no clear experimental proof for this assumption yet, but we consider it to be very reasonable. An immediate consequence of this assumption is that a flat patch requires a stabilizing element to avoid explosive growth, while a curved patch is self-limiting. Here we make our second assumption, namely that the importance of such a stabilizing element should grow with the patch area. Dissociation of triskelia from the area might be such a mechanism, but one also could think about a biochemical one. (2) There is no experimental approach that allows to precisely determine whether the growth angle during the transition from dome to pit is a linear function of time or not, but this is obviously the simplest assumption one can make. (3) The 40:60% ratio is less of an assumption but more of an estimate that follows from the data in conjunction with assumption (2). This ratio has been estimated within the constant area model, the ratio of domes and pits is directly proportional to their transformation dynamics. As such, we have exploited the distribution of domes and pits observed in our EM (40:60%, respectively) to define the time necessary to ultrastructurally rearrange a flat CCS to dome and subsequently to pit. Importantly, we also note that this ratio is in good agreement with the results from Avinoam and coworkers. (4) The exponential decay that was integrated in our mathematical equation of pit growth is a very minor aspect of our work. It is only here to robustly define the time of pinching. We do not extract any information from this exponential decay and we do not use this information to determine whether an object might be flat, curved or a pit. We only use it to define the end-point of a CCS (pinching time).

In the revised manuscript, we now explained these three aspects in more detail. Most changes in this regard are in the text, but for the 40:60% ratio we now also give more information in Supplement info and Supplementary Fig.4. In details:

For assumption (1) where the patch grows: Lines 168-176 and 202-205

For assumption (2) growth angle during transition from dome to pit is a linear function of time: Lines 617-622, 716-722, and 793-798.

For assumption (3) the 40:60% ratio: Lines 183-187, 608-617, and 622-624.

For assumption (4) the exponential decay: Lines 605-607, 765-766

The results of such model, can be used, in my view, for presentation of the experimental data in the language of kinetic parameters, but not for proving the hypothesis.

We completely agree with the reviewer: we also believe that our approach does not prove our assumptions, but it is a novel and self-consistent way to integrate the different data sets into one common framework for analysis. We first used our approach to challenge previously proposed growth models, and could rule out both the constant curvature and constant area models. We then used our equations to obtained the best possible fit and propose an alternative model which is backed up by our plasma membrane tension results.

In response to these comments, we now explain the spirit of our model in more detail when we discuss Figure 3 (Lines 158-163). We also make a comment in this direction in the discussion. (Line 321-325)

The observation that not only the relative amount of curved clathrin structures, but also the amount of clathrin bound AP2 are suppressed by tension is interesting, but leaves a reader with a question of whether AP2 promotes curvature or the curvature promotes AP2 binding.

How the flat to curved transition is regulated remains indeed an open question and we do not claim that we can answer it in this study. However, we do make some important observations in this context. We found that curvature is not necessary to recruit AP2 (we found AP2 on flat structure, compare the CLEM data), or to promote AP2 binding. Indeed, we propose that AP2 reaches its plateau prior to the onset of curvature. Using CLEM approaches, we can observe that the biggest flat structures contain the same amount of AP2 compared to fully formed CCP, while the clathrin content still increases an additional 30% (see figure below). This supports the notion that recruitment of more AP2 is not mandatory for induction of curvature and that acquisition of curvature is not linked to more AP2 recruitment. This is further supported by our observation that under conditions of high plasma membrane tension, clathrin structures are arrested as flat arrays and contain their final AP2 content. Upon restarting of coat dynamics, AP2 content stays the same while only clathrin content increases (Figure 6-7). In the revised manuscript, we now have discussed in more details the change in coat ultrastructure in respects to AP2 levels. Lines 382-389.

Figure: CLEM of CHC and AP2 revealed that the biggest flat structures contain the same amount of AP2 compared to fully formed CCP (Lower panel, b). On the contrary, clathrin-coated pits contain significantly more CHC compare to flat and dome structures (Upper panel, a). This strongly supports our model that the clathrin content still increases during conversion from flat to curved while AP2 remains stable.

**Reviewer #2**

In this work, the authors aim to clarify the ultrastructural rearrangements of the clathrin coat and curvature generation during endocytosis. They challenge the two existing but controversial growth models, constant curvature and constant area, by quantifying the size and components of the clathrin-coated pits (CCPs) in mammalian cells. The quantifications are fulfilled by microscopy (EM, CLEM and TIRF) and mathematical modeling. Through quantitative imaging of EM deep-etch micrographs, the authors present a strong and convincing perspective that challenges both the constant curvature and constant area models by comparing the projected area and surface area of flat, dome and highly curved CCPs. The EM observations lead them to further employ mathematical modeling and life time TIRF imaging of the clathrin/AP2 assembly during clathrin-mediated endocytosis. Based on these data, they argue that the clathrin-coat initially grows flat and starts to bend when it reaches 70% of its final clathrin content, at which time they detect a change in clathrin/AP2 ratio, which the authors identify as a marker of the starting point of curvature developments. In addition, plasma membrane tension is reported to be a key regulator of the curvature change of the clathrin-coat. The combination of quantitative microscopy and mathematical modeling, which nicely fills the gap between EM and live cell imaging in which the desired temporal and spatial resolutions cannot both be satisfied experimentally, is novel. Together these studies provide convincing data eliminating both models. Moreover, they apply their mathematical models to reinterpretation of a previous CLEM study by Kaksonen and Briggs, to reconcile these two studies. Finally, while still not mechanistically understood, the correlation between a drop in AP2:clathrin ratios provides the first potential molecular correlate for curvature.

We are glad to read that reviewer 2 believes that we have provided convincing evidence challenging both constant area and constant curvature models and that he/she believes that correlation between the drop of AP2/clathrin ratio and onset of curvature provides a novel and first potential molecular explanation of coat rearrangement.

Specific major concerns: 1. There are still limitations to both of the clathrin-solo models for curvature generation that should, at least, be discussed. Specifically in Eq(1) in fig.3 yields a good time course (convex curve) but poor geometry distribution. Eq(2) in fig.3 yields exactly the opposite. The concave time course (fig.3(e)), renders Eq(2) potentially worse than Eq(1). First, because the concave time course is controversial, and second, because of the poor match with data points at the beginning of the time course fig.3(e). These early experimental data points are missed by Eq(2) but well captured by Eq(1). These data points appear to cause an artifact that shifts the geometry distribution in fig.3(f).

We thank the reviewer for these very helpful comments. We first note that both growth laws have the same initial regime of accelerated growth, because in both cases we assume growth over the rim. The difference between the two equations is the mechanisms that limit growth: either a mechanism whose importance grows with area (e.g. dissociation from the area), or self-limitation by curvature. Both mechanisms lead to a plateau, but the second model (for the sphere) has a more symmetric time course than the first one (for the patch). The agreement between the experimental time course and the theoretical growth course can be quantified with a chi-squared measure. We find $\chi_{\mathrm{Eq}.2}^{2}/\chi_{\mathrm{Eq}.1}^{2}=0.85$ for the examples shown in Fig. 3b and 3e, thus Eq. (2) actually gives the better time course, not Eq. (1). This clearly shows that one should not over-interpret the time course. The focus of our study is rather the geometry distribution. It is the core idea of our approach to define two models that make sufficiently different predictions regarding the distribution of the different projected areas, so that we can make statements on their dynamical behavior. We also note that due to the limits in resolution, single tracks are not meaningful, but only their statistics. Here we have performed fits with equation 1 and 2 on 4927 tracks extracted from our live fluorescence microscopy of CCS dynamics. As such we can generate strong statistics that fully represent the biological system.

In the revised manuscript, we now have included these explanations (Line 212-216)

For time points located at the beginning of the tracks (white area in Fig.3b and 3e), the computed intensities are below the intensity threshold that has been experimentally measured using CLEM. Objects with intensities equal or inferior to this threshold cannot be detected by EM. These correspond to clathrin objects which are very small (just a few molecules) that can be detected by fluorescence microscopy but cannot be unambiguously identified by EM. They are often masked by unidentified densities and the cytoskeleton. We have now plotted this threshold as dashed line to clarify this in the manuscript (see Fig.3 and Fig.3 legend). We also have modified the projected area schematic to avoid confusion (Fig.3b and e) and annotated our EM detection limit in the manuscript (Lines 187-191). Finally, the region of object that cannot be detected by EM is wider for Eq(2) compared to Eq(1) since the concave region is shorter in Eq1 (see above). Importantly, we do not exclude these points from our computed geometrical distribution according to fitting criteria but because these points will not be detected using our EM approach. This part has been clarified in the supplementary method (Line 721-726)

Disassembly is ignored in Eq(2), which makes it hard to explain the high turnover rate of clathrin in a pit (O. Avinoam, et. al., Science, 2015).

We first note that the turnover measured by Avinoam et al. with FRAP does not necessarily imply that the coat is disassembling. Moreover our first growth law can be interpreted as dissociation, but also in more general terms as some other mechanism coupled to area that limits growth. So in general, we do not believe that the FRAP data is a strong restriction on our model choice, it rather shows the high dynamics of the system and points to possible topological changes, which however we do not address here.

We are well aware that the second growth law neglects disassembly-like mechanisms, but there are two good reasons for this choice. First we note that having both dissociation and limitation by curvature would include two stabilization mechanisms and we would have to make statements about their relative importance, just counteracting our aim to come up with the simplest possible models. Second there is the mathematical issue that a model with both mechanisms would not be able to close the sphere. Although in practise the final stages are dominated by completely different mechanisms anyway (scission by dynamin), here we want to have one simple model that can describe the whole process.

We now understand that this important point needs more explanations and therefore we have now amended the text (202-205) in the supplementary information (Line 747-752).

The authors need to justify the constant rate of angle growth in Eq(2). In sum, it is still unclear whether the ‘updated growth model’, Eq(2) is better than the constant area model, Eq(1), especially when considering the 'concave' and 'koff = 0' flaws in Eq(2). These limitations need to be addressed and discussed, which leads into the need for more input (eg. AP2 model).

As explained above, in our understanding the concave shape is not an issue and the koff=0 assumption is completely in line with our basic reasoning to define two complementary growth laws that both are simple and can be used to fit a large body of experimental data sets. The constant rate of angle growth mentioned in the supplement is a mathematical consequence of the second growth law and not an assumption we make.

2. The model-experiment agreement is much enhanced after the addition of AP2. However, the authors need to justify the usage of 'edge gain, bulk lost' model to describe AP2 kinetics, namely Eq(1). It is obvious that AP2 in the bulk is sandwiched by both the PIP2 on the membrane, clathrin coat and other endocytic accessory proteins (EAP) such as FCHo. Thus, the bulk AP2 could be very stable. The authors should at least discuss this possibility. Ideally, a comparison of FRAP turnover rates of AP2 and clathrin at CCPs, which has not been done, would provide direct evidence for escape of bulk AP2 at the rate predicted by the model.

We first note that as mentioned above, FRAP demonstrates exchange dynamics and not disassembly. We are not aware of any experimental results that do prove or disprove the 'edge gain, bulk lost' assumption. Second, we note that the dissociation term could also be interpreted as any other growth-limiting mechanisms, as long as it scales with area. Third, we note that surprisingly AP2 is highly exchangeable within the clathrin coat. Work from Wu et al., 2003 MBoc, Avinoam et al., 2015 Science and our own data clearly demonstrate that both AP2 and clathrin can be turned-over very efficiently during CCP growth. In the revised manuscript, we now have emphasized the high exchange rate of AP2 to justify our mathematical approach (Lines 804-810).

3. The analysis on the osmotic shock (fig.6 and 7) does not include the clathrin time course, which exhibits a significant increase in the flat portion (compare fig.6(c) to fig.5(e)). The authors need to use a consistent method in this part and discuss these differences.

In unperturbed conditions (isoosmotic), the growth rate of clathrin is positive and defined by the cellular system. On the contrary, around 5 min post-osmotic shock, the clathrin growth rate drops to zero and recovers around 10-12 min post-osmotic shock (Fig 6b). As such, our mathematical model based on clathrin dynamics cannot be applied anymore. We cannot generate a fitted curved for clathrin in osmotic shock conditions as this will require that we could write mathematically how cells respond to osmotic shock (arrest of endocytosis: clathrin growth rate=0) and how they counteract the osmotic shock (restart of endocytosis). Nonetheless, we have controlled that our “ratio histogram” approach (Fig 6) provide us the same ratio of flat, dome and pit CCS under unperturbed conditions than predicted with “fitting and sampling approach ” (Fig 5). This has been highlighted in the text (Line 291-295) and in the supplementary method (Line 845-850)

4. The authors need to quantitatively define 'flat, dome and pit' from EM, and how they are related to the three groups in the model.

We have added a small definition of the flat, dome and pits in the method and clearly stipulate the criteria used to define these three groups (see lines 539-546).

5. Figure 2c, and Figure 1f,g are both obtained from EM imaging but look very different. Also, there is no data from this part that goes into the model. The authors thus need to justify this difference, put the CLEM to better usage, or move it into supplemental.

The CLEM in figure 2 is performed using immunolabelling of clathrin using an anti-clathrin heavy chain antibody. Using this procedure, antibodies create a background density on top of the clathrin coat, making visualization and classification of small clathrin arrays difficult. As such we have an overall over-representation of the pit structures. We have exploited these CLEM data in two instances. First, to validate our findings concerning when flat structure acquire curvature (70% of clathrin content). From our CLEM data, we can indeed observe that the fluorescence intensity of the large flat structure represents 70% of the intensity of fully form pits. Second, we have used these CLEM data to correlate fluorescence intensity and projected area which we have exploited in figure 4 to conclude that AP2/clathrin ratio in a CCS differs depending on its curvature and that this ratio is reduced within the coat as curvature increases. The above statements were in the supplementary information but we now have updated the text to clarify the importance of the CLEM data sets (Lines137-139, 160-163).

6. The authors need to show the distributions of the fitted parameters (in appropriate units), making sure they are in reasonable ranges. Especially kon and koff.

We added the mean values of the fitted parameters to the manuscript as in table 1 (k_on can be calculated from the other parameters) and provided you below the distributions. (Lines 698-700 and 780-781)

7. The AP2 -> clathrin feedback is oversimplified as a one-time change to the parameter t_transform, when the flat coat gains curvature. However, AP2 obviously regulates clathrin coat formation in the earlier phase. The authors need to consider conventional models that treat AP2 and clathrin as dynamically interacting variables, and to fully capture the AP2 -> clathrin feedback as a Hill function, replacing the one-time change.

8. The authors need to justify ignoring clathrin -> AP2 feedback.

We agree that there is a molecular feedback between clathrin and AP2, and ultimately this could be integrated in a proper molecular dynamic model of CCP formation, where binding hierarchy, kinetics, structural changes of AP2 and clathrin and other adaptor and accessory proteins should be integrated. Here, our approach was not to perform a molecular dynamic modelling of CCP formation but simply to provide simple mathematical models of CCP growth profiles. We only describe the transition from flat to curved in a geometrical sense and not in a molecular sense. As such, we use the time offset (time of AP2/clathrin ratio change) as a putative time of ultrastructural rearrangement. We challenged this hypothesis by calculating the predicted distribution and comparing it to the distribution observed by EM. This approach is further confirmed by our osmotic shock experiments.

9. 24 second lifetime threshold is too long. The authors at least need to show, for example, 12 second threshold would not affect the model and the conclusions significantly. Also, it is strongly suggested to test whether using maximum intensity threshold instead of lifetime threshold could lead to better results.

We now have performed our analysis using a 12 second threshold. As you can see below we found no significant difference in the size distribution of flat, domes, and pits compared to fig 3c, 3f and 5g, which were generated with a 24 second lifetime threshold. Additionally, we performed a Welch’s t-test to compare 12 sec- to 24 sec threshold for the data set of one cell and found no significant difference (Fig. 3c: p = 0.00004, Fig. 3c: p=0.005, Fig. 5g: p=0.07). We think that the small differences come from the difference in the size of the data set that we used to calculate the geometrical distribution (from all fitted tracks we extract more than 4 million time points). We have added a comment in the text (lines 677-681) but decided not to include this new figure with the 12 sec threshold in the manuscript.

Concerning the usage of maximum intensity threshold instead of lifetime threshold. This is an interesting idea which we also considered during our analysis. However, it turned out that such an intensity threshold could potentially affect our results in a much more “uncontrollable” manner. Indeed, from cell to cell, the fluorescence intensity can be very different and as such this maximum intensity threshold will have to be redefined for each cell. On the contrary, we have observed that the lifetime of clathrin structures varies less from cell to cell and therefore represent a more robust parameters.

10. The error bars in Fig.1(g) are too big, especially near the mode. Analyzing several more EM figures is strongly suggested to reduce the error, which could otherwise greatly affect the model fitting.

We now have analyzed more membranes in EM. The error bars, which show the standard deviation, did get a little smaller, but unfortunately, the standard deviation remained relatively high. This broad distribution represents the cellular variability of CCS that can be seen from cell to cell. Overall the outcome from 3 or 6 analyzed membranes are similar, see candle plots below. Variations are stronger for smaller objects due to the detection limit in EM and sample quality (more actin can cover small coats).

11. At some point in the text, it should be noted that the cells are plated on poly-l-lysine. The possible effects of ‘gluing’the cells to glass should not be ignored. Certainly, this makes a difference with regard to actin dependence for endocytosis (see Yarar et al., 2004, PMID: 15004222)

All experiments in this work (FM and TEM) were performed on poly-D-lysine (PDL), which is necessary for the EM technique. We made sure that this was clearly stated in the method section. We have tested various “coating” proteins and monitored their impact on pits dynamics. We did not find significant differences in the lifetime of CCS when comparing PDL, collagen and “naked” glass. We only observed minor differences when seeding cells on fibronectin-coated coverslips (see below). BSC-1 cells seeded on poly-L-lysine did not attached very well on the substrate and display morphology indicative of cytotoxic effect. We decided to not use poly-L-lysine for this reason.

Minor concerns:

1. Need to show quantitatively how 'multiple maximum tracks' are filtered out, and how the filtering can shift the data.

We have added a description to the Supplementary Material. Lines 684-688

2. It is unclear why there are no multiple structures (which is presented in Figure 2) in Figure 1f,g. The authors need to justify this difference.

Multiple structures in CLEM are clathrin structures that can be distinguished with the resolution of EM (e.g. a flat structure close to a pit) but not with the resolution of FM. They only appear in the CLEM analysis and not in the EM analysis, because we can detect fluorescent objects by FM that appear as one signal but when using CLEM to look at them by EM, they appear as multiple object close to each other (not a single CCS). Definition of multiple structures in CLEM has been be added to the main text (lines 129-132) and illustrated in Fig.2a.

4. Line 98: define 'TEM'. Added

5. Line 594: typo.  Changed

6. Line 340: typo.  Changed

7. Line 672 and Eq(2): justify the unit of t. Changed. Lines 760-761

The manuscript was revised and transferred to Nature Communications. You will find below the reviewer comments after the transfer and our answers to them.

**To reviewer #1:**

The authors convincingly addressed my previous questions and comments and I am glad to recommend publication of the revised manuscript in Nature Communication

Thank you for accepting our manuscript, we are very happy that our changes met with your approval.

**To reviewer #3:**

The authors use a combination of high end imaging techniques and mathematical modelling to study the early stages of clathrin-coated endocytosis, specifically around the formation and growth of clathrin coated structures. From their imaging data they argue that 2 existing competing models can not describe their findings and they propose a new model which is mainly built on modelling their experimental data. The way they have conducted the experiments and built on their experimental data is technically sound and supports their findings. As argued by one of the reviewers of the initial manuscript it would be ideal if this new model could be tested in a robust way. However I do believe that the manuscript provides a significant step forward in our knowledge and would warrant publication, also for other groups to test this new hypothesis and potentially design this robust test. I believe they have satisfied most of the comments from the previous rounds and that this is a much improved version of the manuscript so it would be unfair to completely restart the reviewing cycle.

I only have minor comments left for improvement of the manuscript, some of which are simple spelling errors:

- I could not find a thorough explanation how the fluorescence is quantitated. This is one of the critical data sets of the paper so it would be good to have a clear description of this.

- line 166: according to

- line 174: such a process

- line 382: not linked

- line 384: be involved

- line 533: and then carefully

Figure 8: the top part of the figure does not match the relative distribution of AP2 vs clathrin. This should be corrected with less AP2 in the later parts of the top figure I assume.

Suppl. Fig.2 I was surprised to see donut-shaped cathrin-coated profiles in the STED microscope. Please explain.

Thank you for taking the time to review our manuscript and we really appreciate your comment that our work represents a significant step forward in the field. We have addressed your minor point as follow:

-We have now better described how quantification of fluorescence was performed in this work. See text line 532, 548, 569

-The spelling mistakes have been corrected. See text line 174, 182, 399, 401, 526

-Figure 8 has been modified and the number of AP2 is now identical at the beginning and end of curvature rearrangement which is the important message of our model. Thanks for noticing this mistake.

-Due to the spherical shape of clathrin-coated pits, we find an accumulation of signal at the rim of such a structure compared to the top. As a matter of fact, there is signal in the middle of the donuts (which corresponds to the top of the clathrin-coated pit) but it is less compared to the rim region. As such, this results in a donut shape. Importantly, the impression of a donut-shaped clathrin structure is not a result from processing or deconvolution since these structures are clearly visible in the raw data.

**To reviewer #4:**

I was asked to review the article entitled, ‘Flat to curved transition during clathrin-mediated endocytosis correlates with a change in clathrin-adaptor ratio and is regulated by membrane tension’ in lieu of the original reviewer 2 and see if his/her comments were addressed in the revision. I will begin by highlighting that the paper has a remarkable amount of experimental work that is of high quality. A majority of Reviewer 2’s points were focused on the model formulation, assumptions, and goodness-of-fit with respect to the dynamics. In reading the manuscript to assess if these concerns had been addressed, I found that the mathematical model was sorely lacking in any mechanisms that would explain how the area was growing. And that is my biggest reservation with the study. I appreciate the authors’ using the words growth laws but there is no evidence to suggest that we can model the growth of the coat as a purely kinetic mechanism. At the very least, since the rate law has to account for clathrin from bulk solution or cytoplasm binding to the membrane, it involves a surface reaction. That makes the kinetics geometry dependent. Therefore, I feel that the modeling component of the study is the weakest part. At best, it affords a fitting to the observed dynamics and not a great one. While the authors have provided a table to demonstrate goodness-of-fit for model parameters it is not explained what these parameters mean or how they are relevant. I will note that the original comment 7 about feedback was not incorporated in the model. And finally, my most major reservation -- the authors perform experiments with osmotic shock to show how tension affects the flat-to-curved transition and assert that these experiments validate the model (lines 313-315). How? The models do not contain any tension dependent terms. I believe that the authors would benefit from removing the kinetic model from the paper and focusing more on the experiments. A model that relates mechanics to dynamics must be better developed than the one presented here and merits a study of its own. As it stands, I cannot recommend the manuscript for publication in its current form.

Thank you for accepting our manuscript, we are very happy that our work met with your approval.

In response to reviewer 4 comments: Their main reservation was the lack of any mechanism which would explain how the clathrin-coated area was growing. Our approach was not to create a complex model with molecular details but rather to create a simple mathematical description of CCP growth. There are several models used in the field to describe how the clathrin coat can bend or not bend during growth and these approaches, although very informative they did not yet solve the issue. Our approach is completely different and therefore novel: rather than coming up with a complex model, we take very simplistic growth laws which have the minimally required terms for growth of flat versus bent coats. Importantly, they can be fitted easily to a huge amount of data sets obtained using live confocal microscopy to estimate the transition point from flat to bend. Our idea was to describe the growth of a CCP from initiation to full formation and to also describe the transition from flat to curved in a geometrical sense. For this, assumptions were made, such as the transition from domed to pit is a linear function of time. We agree with reviewer #4 that a model that relates mechanics to dynamics will provide a valuable description of CCP growth but as he/she noted this would constitute a study on its own. Importantly, the three main conclusions of our work (1) Neither the constant area model nor the constant curvature model can explain the biological distribution of the various CCP structures, (2) The transition from flat to curved is marked by a change in the clathrin/AP2 ratio, (3) Membrane tension suppresses the flat to curved transition, have all been determined experimentally and are not the direct conclusion of our model. The model here is mostly used to drive the work forward as a means to provide a statistical distribution of the various clathrin structures and their size/projected area (pit, dome, flat). We used this mathematical approach to fit the growth kinetics that we have obtained from our confocal microscopy to mathematical equations that describe putative growth models. This allowed us to compare predicted distributions (from putative and simplified model) to the real measured distribution of the various clathrin structures (EM and CLEM). Although our mathematical description is a very simplified model, it still useable and sufficient for such direct comparison and allows us to estimate multiple parameters that were verified experimentally (the 40:60% ratio, the change in clathrin/Ap2 ratio and the arrest of flat structures upon increased membrane tension).

Concerning the osmotic shock and membrane tension, the reviewer is right our model does not contain tension dependent terms. We only fitted the growth kinetic profiles obtained using confocal microscopy under osmotic conditions to our mathematical description of pit growth. This allowed us to predict that the stalled objects will be arrested as flat clathrin arrays which was then experimentally verified using EM of metal replica. We agree that the statement we made in line #330, should be tuned down. We have changed it to “these findings are in agreement with our model describing that the change in clathrin/AP2 ratio represents the precise moment at which the flat-to-curved transition occurs”.

In regards to the goodness of fit of our model, we decided for a fitting approach based on the basis of distribution rather than on the basis of single trajectories. Therefore, the goodness-of-fit of a single fit is not key to our decision but it is the goodness-of-fit of the distribution between calculated distribution and measured histograms that allows us to test hypothesis and challenge the various growth models. We use p-values in the main text and chi-square in the supplement. This information was in the figure legends but the rational of test choice and their consequence could be easily better explained in the text to avoid confusion.

Concerning the original comment number 7, we addressed this reviewer comment together with comment 8 in our reply letter and indeed, we did not incorporate feedback between AP2 and clathrin because, as mentioned above, our strategy was not to create a complex model with molecular details including dynamics, mechanistic and feedback mechanisms but a simple mathematical description of the distribution of the various clathrin structures.

In conclusion, we agree that our model is a simple geometric description of pit growth but again we want to emphasize that our findings arose from experimental data and are not the results of a prediction. The reviewer #4 themselves highlights that the paper has a “remarkable amount of experimental work that is of high quality”. We have the feeling that reviewer #4 agrees with our biological conclusions but simply had a different expectation of the complexity of our modeling approach. Reformulating our approach to create a full molecular dynamic model of clathrin coat growth will be very time consuming and hardly possible with the knowledge and experimental methods currently available (for example, we do not have atom-level or cryoEM information that precisely describe which conformational changes take place when the clathrin lattice bend from flat to curve structure. To make such a model, many assumptions would have to be taken making the more complex model also imperfect). Moreover, we hope that we have convinced you that fully changing the mathematical model will not change the biologically generated conclusion of our work.
